# Functional genomics of RAP proteins and their role in mitoribosome regulation in *Plasmodium falciparum*

**DOI:** 10.1038/s41467-022-28981-7

**Published:** 2022-03-11

**Authors:** Thomas Hollin, Steven Abel, Alejandra Falla, Charisse Flerida A. Pasaje, Anil Bhatia, Manhoi Hur, Jay S. Kirkwood, Anita Saraf, Jacques Prudhomme, Amancio De Souza, Laurence Florens, Jacquin C. Niles, Karine G. Le Roch

**Affiliations:** 1 Department of Molecular, Cell and Systems Biology, University of California Riverside, Riverside, CA USA; 2 Department of Biological Engineering, Massachusetts Institute of Technology, Cambridge, MA USA; 3 Metabolomics Core Facility, University of California, Riverside, CA 92521 USA; 4 Stowers Institute for Medical Research, 1000 E. 50th Street, Kansas City, MO 64110 USA

**Keywords:** Parasite biology, RNA metabolism

## Abstract

The RAP (RNA-binding domain abundant in Apicomplexans) protein family has been identified in various organisms. Despite expansion of this protein family in apicomplexan parasites, their main biological functions remain unknown. In this study, we use inducible knockdown studies in the human malaria parasite, *Plasmodium falciparum*, to show that two RAP proteins, PF3D7_0105200 (*Pf*RAP01) and PF3D7_1470600 (*Pf*RAP21), are essential for parasite survival and localize to the mitochondrion. Using transcriptomics, metabolomics, and proteomics profiling experiments, we further demonstrate that these RAP proteins are involved in mitochondrial RNA metabolism. Using high-throughput sequencing of RNA isolated by crosslinking immunoprecipitation (eCLIP-seq), we validate that *Pf*RAP01 and *Pf*RAP21 are true RNA-binding proteins and interact specifically with mitochondrial rRNAs. Finally, mitochondrial enrichment experiments followed by deep sequencing of small RNAs demonstrate that *Pf*RAP21 controls mitochondrial rRNA expression. Collectively, our results establish the role of these RAP proteins in mitoribosome activity and contribute to further understanding this protein family in malaria parasites.

## Abstract (web summary)

The function of RNA-binding domain abundant in Apicomplexans (RAP) protein family members is largely unknown. Here, using high-throughput functional genomics, including metabolomics, Hollin *et al*. characterize two RAP proteins that are essential for *Plasmodium falciparum* survival and control mitochondrial rRNAs.

## Introduction

RNAs associate with RNA-binding proteins (RBPs) to regulate a wide range of essential processes in eukaryotes. Diverse RNA-binding domains (RBDs) have been identified, including the RNA-recognition motif, zinc finger, K Homology domain, and Pumilio homology domain^1,2^. These domains are involved in a variety of cellular processes including splicing, mRNA processing, stability, and translation^3^. While several RBDs are well conserved in eukaryotes, some domains have species-specific roles. This is particularly true for a domain known as RBD abundant in Apicomplexans or RAP. This domain was originally described as specifically enriched in apicomplexan genomes with 11–15 members in *Plasmodium* spp. and *Toxoplasma gondii*, while only 4–6 members (annotated as FASTK) were identified in mammalian genomes^4^. Recent studies reported that the enrichment is even higher than initially thought, with 21–23 RAP proteins in the apicomplexan parasites^5–7^. The exact function of the RAP proteins remains to be determined. In eukaryotes, RAP proteins have been predicted to be involved in RNA binding^4^ and seem to be linked to organelle function since most of the characterized proteins have been located in mitochondria^8,9^ or chloroplasts^10–12^. Several reports in model organisms have also confirmed their role in RNA metabolism in humans and plants^9,10,12–17^. Structurally, RAP proteins are divided into a variable N-terminal region composed of helical repeat protein motifs and the RAP domain on the C-terminal side^7^. These repeated sequences are stacked together and form a superhelix with an RNA-binding groove^18,19^. They have been identified in some RAP proteins as Heptatricopeptide repeat (HPR) motif^20^, structurally and functionally related to Octotricopeptide and Pentatricopeptide repeat (PPR) motifs. Most of these proteins are predicted to target organelles and play different roles in RNA metabolism and translation^12,18–21^. Regarding the RAP domain, the conserved part is composed of ~60 amino acids and has an α/β topology, similar to restriction endonuclease-like folds, with multiple aromatic and charged residues^4,7,14^.

*Plasmodium falciparum*, the deadliest human malaria parasite, possesses 22 potential RAP proteins^7^, 18 of which are putatively essential for the asexual parasite survival^22^, indicating the crucial role of this family. Despite the potential of these proteins as novel therapeutic targets, they remain poorly characterized. Experimental capture of RBPs in the blood stages of *P. falciparum* using oligo d(T) beads captured 199 proteins including two predicted RAP proteins, PF3D7_0105200 (*Pf*RAP01) and PF3D7_1470600 (*Pf*RAP21)^5^. Here, using transgenic lines in which *Pf*RAP01 and *Pf*RAP21 expression was conditionally regulated, we validated the essentiality of these proteins and showed their localization to the parasite mitochondrion. Furthermore, using transcriptomics, metabolomics, and proteomics profiling experiments, we demonstrated that down-regulation of these RAP proteins affects distinct RNA biology and mitochondrial processes. Finally, using high-throughput sequencing of RNA isolated by crosslinking immunoprecipitation (eCLIP-seq) and mitochondrial enrichment followed by sequencing of small RNAs, we confirmed these proteins are true RBPs that bind mitochondrial rRNAs in situ, thus validating their role in parasite mitoribosome metabolism.

## Results

### Generation of PF3D7_0105200 (*Pf*RAP01) and PF3D7_1470600 (*Pf*RAP21) transgenic lines

Comparative protein sequence analysis revealed that PF3D7_0105200 (*Pf*RAP01) is highly conserved across *Plasmodium* spp., with 72% and 76% of identity with its homologs in *P. vivax* and *P. berghei*, respectively (Supplementary Data 1). PF3D7_1470600 (*Pf*RAP21) is also conserved in *Plasmodium* spp., but has lower sequence identity with *P. vivax* (38%) and *P. berghei* (38%).

**Fig. 1:**
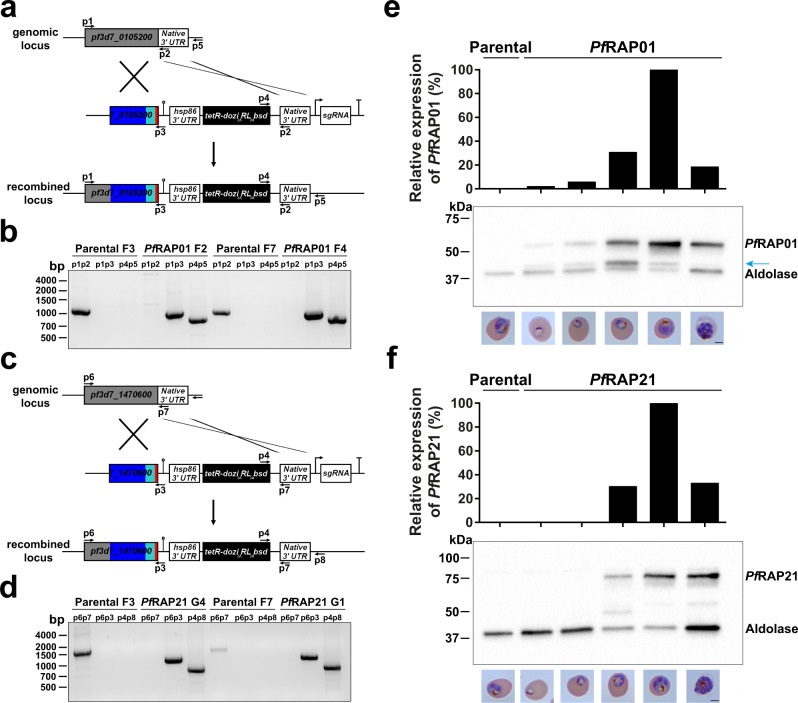
Validation of *Pf*RAP01 and *Pf*RAP21 transgenic lines. Schematic representation of the TetR-aptamer integration strategy into endogenous *pf3d7_0105200* (**a**) and *pf3d7_1470600* (**c**). TetR-dozi-BSD cassette (black), HA-tag (red), PCR primers, homologous regions used (blue) and recodonized sequences (light blue) are indicated. Genotype analyses of parental, *Pf*RAP01 (**b**) and *Pf*RAP21 (**d**) clone lines. PCRs were performed on genomic DNA from indicated lines using different primer combinations. The PCRs are representative of three independent experiments. Immunoblot detection of *Pf*RAP01 (**e**) and *Pf*RAP21 (**f**) expression across the asexual blood cycle. The membranes were probed with anti-HA and anti-aldolase was used as loading control. The expression of *Pf*RAP01 and *Pf*RAP21 were normalized by aldolase expression. The blue arrow indicates *Pf*RAP01 degradation. Scale bar: 2 μm. The immunoblots shown are representative of two independent experiments.

Previous failed attempts to disrupt *Pf*RAP01 and *Pf*RAP21 in *P. falciparum*^22^ and their respective homologs in *P. berghei*^23^ suggested that both proteins are essential. To gain insights into the function of these proteins, we used an inducible knockdown based on the TetR-DOZI/RNA aptamer system, in which translation levels of the target protein is controlled by anhydrotetracycline (aTc)^24–26^. The lines were generated using CRISPR/Cas9 and homology directed repair in the NF54::pCRISPR^INT^ line with *T7 RNA polymerase* and *SpCas9* integrated at the *cg6* locus^25^ (Fig. 1a, c). *Pf*RAP01 and *Pf*RAP21 were modified to include a C-terminal 3x-HA tag during the genome editing step used to install the regulatory components needed to achieve conditional expression. Clonal lines, *Pf*RAP01 F2 and F4 for PF3D7_0105200, and *Pf*RAP21 G1 and G4 for PF3D7_1470600, were obtained by limiting dilution and used in subsequent studies. The expected integration events in the *Pf*RAP01 and *Pf*RAP21 loci were validated by PCR (Fig. 1b, d). We also performed whole-genome sequencing of the parental, *Pf*RAP01, and *Pf*RAP21 clones and confirmed the correct insertion of the inducible system (Supplementary Fig. 1). No major deletions or duplications were detected. Mutations in *var* genes and a nonsense mutation in *ap2-g* were observed in the parental, *Pf*RAP01, and *Pf*RAP21 clones, validating the absence of off-target editing. The *ap2-g* mutation is consistent with lack of gametocyte production in all of these parental and transgenic cell lines.

We verified the expression of *Pf*RAP01 and *Pf*RAP21 across the intraerythrocytic development cycle (IDC) by Western blot analysis using the 3x-HA tag (Fig. 1e, f). We observed a peak of expression at the mature trophozoite stage for both RAP proteins, in correlation with the RNA expression profiles available^27–29^. Collectively, these data confirmed successful generation of *Pf*RAP01 and *Pf*RAP21 transgenic lines.

### Mitochondrial localization of *Pf*RAP01 and *Pf*RAP21

**Fig. 2:**
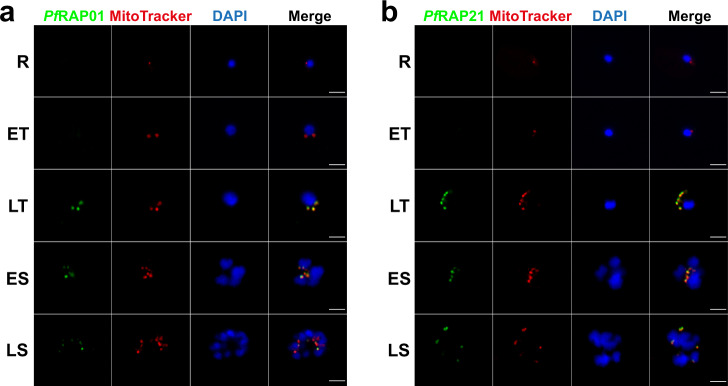
Localization of *Pf*RAP01 and *Pf*RAP21 in asexual blood stages of *P. falciparum*. Immunofluorescence assays of *Pf*RAP01 (**a**) and *Pf*RAP21 (**b**) on ring (R), early trophozoite (ET), late trophozoite (LT), early schizont (ES) and late schizont (LS) stages. Both proteins were labeled with anti-HA and co-localized with parasite mitochondria stained with MitoTracker. Merge shows HA tag, MitoTracker and DAPI signals. Scale bar: 3 μm. The IFAs are representative of three independent experiments.

In accordance with the predominant localization of RAP proteins to the mitochondria or plastid, *Pf*RAP01 and *Pf*RAP21 have been predicted to localize to the parasite mitochondrion (Supplementary Data 1). To validate this prediction, we performed anti-HA immunofluorescence assays (IFAs) to detect the 3x-HA tagged *Pf*RAP01 and *Pf*RAP21 proteins. We showed that *Pf*RAP01 and *Pf*RAP21 both co-localized with MitoTracker, a red-fluorescent dye that stains mitochondria in live parasites (Fig. 2a, b). For *Pf*RAP01, this is consistent with the mitochondrial localization of the *P. berghei* ortholog, PBANKA_0208100^20^. The lack of *Pf*RAP01 and *Pf*RAP21 detection by IFA in rings and early trophozoites correlates with their respective expression across the asexual cycle (Fig. 1e, f). The lack of HA-signal on the parental line confirmed the specificity of these IFAs (Supplementary Fig. 2a).

Using an anti-Cpn60, an apicoplast chaperonin^30,31^, IFAs revealed a distinct localization between the apicoplast and *Pf*RAP01 or *Pf*RAP21 indicating that they are not directly associated with the plastid (Supplementary Fig. 2b). Pearson’s coefficient confirmed this result with a mean score at 0.31 and 0.84 for Cpn60 and MitoTracker signals, respectively (Supplementary Fig. 2c).

### Essentiality of *Pf*RAP01 and *Pf*RAP21 in erythrocytic cycle

**Fig. 3:**
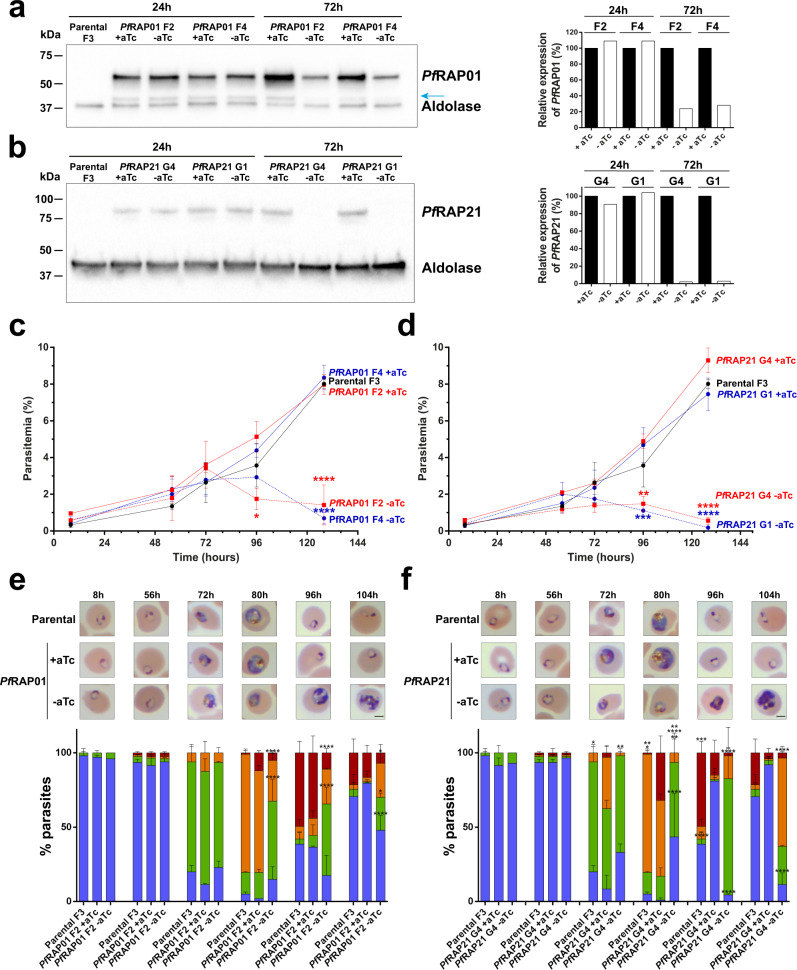
Essentiality of *Pf*RAP01 and *Pf*RAP21 in asexual blood stages. Immunoblot detection of *Pf*RAP01 F2 and *Pf*RAP01 F4 (**a**), *Pf*RAP21 G1 and *Pf*RAP21 G4 (**b**) expression in presence or absence of aTc at 24 and 72 h. The membranes were probed with anti-HA and anti-aldolase was used as loading control. The immunoblots are representative of two independent experiments performed. The expression of *Pf*RAP01 and *Pf*RAP21 were normalized by aldolase expression and +aTc condition was considered as 100%. The blue arrow indicates *Pf*RAP01 degradation. Parasitemia of Parental F3, *Pf*RAP01 F2 and *Pf*RAP01 F4 (**c**), *Pf*RAP21 G1 and *Pf*RAP21 G4 (**d**) was measured in presence or absence of aTc. Parasitemia was assessed on ten fields and in duplicate. The results of one representative experiment out of two, are shown as the mean parasitemia ± SEM (Two-way ANOVA and Tukey’s multiple comparison test, *p < 0.05, **p < 0.01, ***p < 0.001 and ****p < 0.0001 compared to clones +aTc at the same time point). Phenotypic analysis of Parental F3, *Pf*RAP01 F2 (**e**) and *Pf*RAP21 G4 (**f**) lines. Percentages of rings (blue), trophozoites (green), late trophozoites (orange), and schizonts (red) are indicated for each time point (*n* = 51–106 parasites counted, mean ± SD). Times indicated correspond to hours post aTc removal. (Two-way ANOVA and Tukey’s multiple comparison test, *p < 0.05, **p < 0.01, ***p < 0.001 and ****p < 0.0001 compared to clones + aTc at the same time point). Scale bar: 2 μm.

Constitutive knockouts of *Pf*RAP01, *Pf*RAP21, and their respective orthologs in *P. berghei* have been previously unsuccessfully attempted, suggesting essentiality of these proteins during asexual blood stages^22,23^. Therefore, we took advantage of our transgenic lines and verified the efficiency of our inducible system by Western blot analysis (Fig. 3a, b). Beginning with synchronous ring-stage cultures, we observed that *Pf*RAP01 and *Pf*RAP21 expression remained significantly stable at 24 h post aTc removal (trophozoite from the first IDC) (Fig. 3a, b). After 72 h (trophozoite from the second IDC), which corresponds to the beginning of the transcription (Fig. 1e, f), we detected a significant reduction in protein levels for both *Pf*RAP01 and *Pf*RAP21 with a reduction of 77% and 97% respectively when compared to lines cultured with aTc. We then performed IFA studies to simultaneously detect RAP levels (anti-HA) and mitochondrial labeling (MitoTracker) at 72 h post aTc removal. We observed a decrease of 76% and 87% of double positive cells for *Pf*RAP01 and *Pf*RAP21, respectively (Supplementary Fig. 3a–d).

To study further the kinetics of parasite growth upon *Pf*RAP01 and *Pf*RAP21 depletion, tightly synchronized ring-stage parasites (*t* = 0 h) were maintained either with or without aTc. We observed no change in parasitemia during the first IDC for the *Pf*RAP01 knockdown compared to the parental and control lines (Fig. 3c). This result was confirmed by the absence of phenotypic change in the first 56 h and ability of parasites under knockdown conditions to reinvade red blood cells with the same efficiency as the controls (Fig. 3e). At 128 h, however, a substantial reduction in parasite proliferation was observed for the RAP-deficient parasites. Microscopic analysis showed that majority of these parasites were blocked in the trophozoite stage during the second IDC (80 h) and began to die while the control parasites continued schizogony and were able to reinvade indicating that *Pf*RAP01 is essential for the IDC. Similar results were obtained upon *Pf*RAP21 knockdown. The parasitemia and developmental stages remained identical throughout the first IDC (Fig. 3d, f). Between 72 and 80 h, corresponding to the transcription peak, parasites were mainly blocked at the trophozoite stage and quickly lost viability resulting in a substantial drop in parasitemia. The changes observed with *Pf*RAP21 appear to be even more drastic than those obtained with *Pf*RAP01, which is consistent with the different knockdown efficiencies detected by Western blot analysis (Fig. 3a, b). Altogether, these data confirmed the independent essentiality of *Pf*RAP01 and *Pf*RAP21 for *P. falciparum* survival. Since clones behaved identically in these studies, the following experiments were performed using only the *Pf*RAP01 F2 and *Pf*RAP21 G4 clonal lines.

To determine if the effects resulting from depletion of RAP proteins during the first IDC can be reversible, *Pf*RAP01 and *Pf*RAP21 parasites were replenished in aTc at 32, 56, or 72 h after initial aTc removal at 0 h (Supplementary Fig. 3e). Quantification of parasitemia using SYBR Green assays at 80 and 128 h showed that the deficiency in *Pf*RAP01 and *Pf*RAP21 can be completely reversed until 56 h (Supplementary Fig. 3f, g). At 128 h, a partial recovery was only obtained for *Pf*RAP01 after a replenishment at 72 h, confirming the more drastic depletion for *Pf*RAP21 as previously shown. This result indicates that no irreversible effects were suffered by the parasites until 56 h leading to complete recovery and pursuit of the IDC.

### Transcriptomic analysis of *Pf*RAP01 and *Pf*RAP21 knockdown parasites

**Fig. 4:**
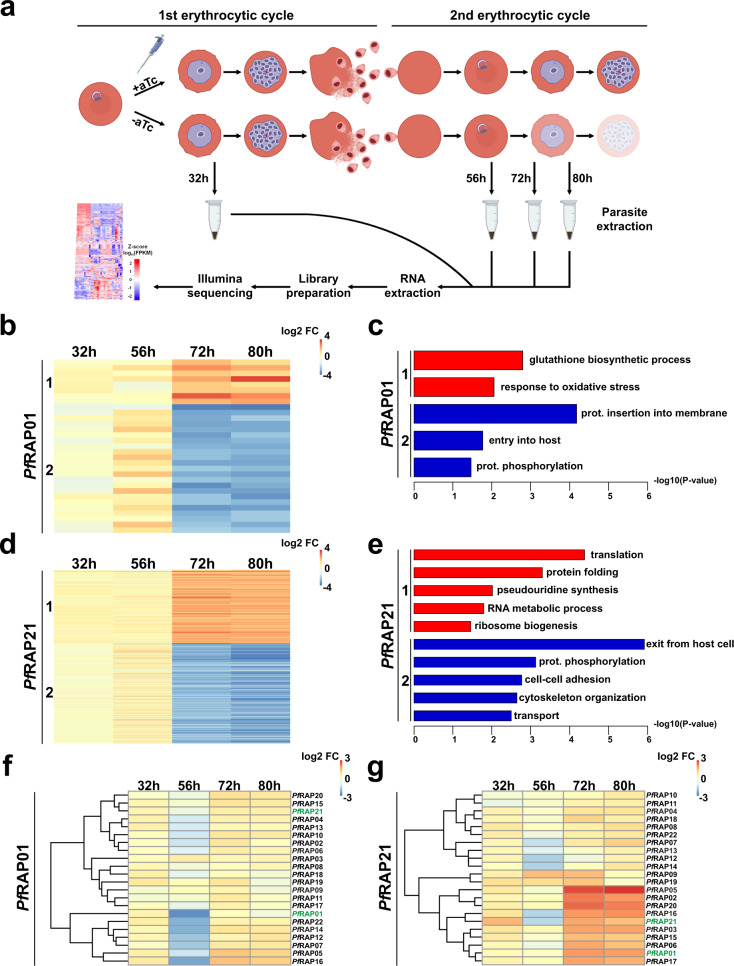
Transcriptome profile of *Pf*RAP01 and *Pf*RAP21 knockdown parasites. **a** The schematic illustrates the key steps of the RNA-seq protocol. Created with BioRender.com. **b**–**d** Clustering of significantly affected genes from *Pf*RAP01 (**b**) and *Pf*RAP21 (**d**) knockdown parasites. Clusters 1 and 2 regroup genes significantly up-regulated and down-regulated respectively (two-tailed Wald test with Benjamini–Hochberg adjustment). **c**–**e** GO enrichment analysis of genes with −log10 (*p* value) for clusters 1 (red) and 2 (blue) (weight01 Fisher test). Heatmaps of the log2 FC values of predicted RAP proteins from *Pf*RAP01 (**f**) and *Pf*RAP21 (**g**) knockdown samples.

To explore the global transcriptomic effects of *Pf*RAP01 and *Pf*RAP21 knockdowns, we performed RNA-seq on the parental line, and the *Pf*RAP01 and *Pf*RAP21 knockdown lines with and without aTc. We selected four time points corresponding to trophozoites from the first IDC (32 h) as well as the rings (56 h), trophozoites (72 h), and trophozoites/schizonts (80 h) from the second IDC (Fig. 4a). Two biological samples were generated for each condition. Spearman correlation coefficients ranged between 0.81 to 0.96 for each replicate, demonstrating the reproducibility of our experiment (Supplementary Data 2). Differentially expressed genes were then identified using the DEseq2 software (see “Methods” for further details). Comparative analysis between the parental and *Pf*RAP01 + aTc or *Pf*RAP21 + aTc lines showed that almost no genes were affected (median = 13.5 genes) during the first and second IDCs, confirming that genetic modification of the RAP loci did not grossly alter the parasite’s transcriptome (Supplementary Data 2). However, this was not the case at ring stage (56 h) of the second IDC, wherein comparison of the parental and RAP + aTc lines revealed a significantly higher number of differentially expressed genes. Gene Ontology (GO) terms enrichment analysis indicated that most of these genes were involved in invasion, suggesting a potential phase shift in cell cycle between the different lines used.

We then analyzed *Pf*RAP01 and *Pf*RAP21 samples in presence and absence of aTc and grouped the genes based on whether they were significantly differentially expressed at 72 and 80 h (Fig. 4b, d). Significantly up-regulated genes were placed in cluster 1 and down-regulated genes were in cluster 2 (Supplementary Data 2). For *Pf*RAP01 knockdown, only eight genes were up-regulated in cluster 1 (Fig. 4b). Although GO terms enrichment analysis indicated that glutathione biosynthetic process and response to oxidative stress pathways were significantly up-regulated, the low number of impacted genes was a hindrance to reaching any conclusion (Fig. 4c). This low significance likely results from a combination of filtering stringency and incomplete down-regulation (77%) of *Pf*RAP01 (Fig. 3a). Cluster 2 contained 23 down-regulated genes, many of which are known to have a role in host-pathogen interactions and invasion. It is highly likely that these genes are identified due to the cell cycle arrest phenotype observed upon *Pf*RAP01 knockdown and are not directly related to the *Pf*RAP01 function. For *Pf*RAP21, 392 genes were described as significantly up-regulated at 72 and 80 h (Fig. 4d). These genes are mainly associated with translation and RNA processing, such as the RNA helicases DBP1/7/8, mRNA-decapping enzymes DCP1 and DCP2, and multiple ribosomal proteins (Fig. 4e). While some of these retained transcripts could be associated with the cell cycle arrest, our result suggests a potential compensatory mechanism of the parasite in pathways affecting RNA biology and translational regulation. Interestingly, we detected 10 mRNAs for RAP proteins as up-regulated in *Pf*RAP21 knockdown parasites at 72 and 80 h (Fig. 4g), including *Pf*RAP01. Such enrichment could confirm a compensatory mechanism, albeit insufficient, of the RAP regulatory network to promote parasite survival upon knockdown of *Pf*RAP21. A similar trend, to a lesser extent, was also observed for the *Pf*RAP01 knockdown line (Fig. 4f). Similar to *Pf*RAP01, cluster 2 of the *Pf*RAP21 samples (528 genes) contains transcripts related to host-pathogen and invasion processes (Fig. 4e).

Although not all statistically significant, the three mitochondrial protein-coding genes were detected with a median log2 Fold Change (FC) at −1.7 and −2.5 for *Pf*RAP01 and *Pf*RAP21, respectively (Supplementary Data 2), indicating an important impact on mitochondrial transcriptional regulation. This disturbance seems to have a less significant impact on nuclear genes predicted to be imported into the mitochondria^32^, since only 40 genes out of 295 were differentially expressed at 72 and 80 h for *Pf*RAP21. As the transcription of these genes is under control of the nucleus, the RAP proteins are probably not directly involved in their regulation, unlike mitochondrial genes. Taken together, these data confirm a significant cell cycle arrest in *Pf*RAP01 and *Pf*RAP21 knockdowns and a possible association with RNA metabolism and translation for *Pf*RAP21.

### Metabolic perturbation of *Pf*RAP01 and *Pf*RAP21 knockdown parasites

**Fig. 5:**
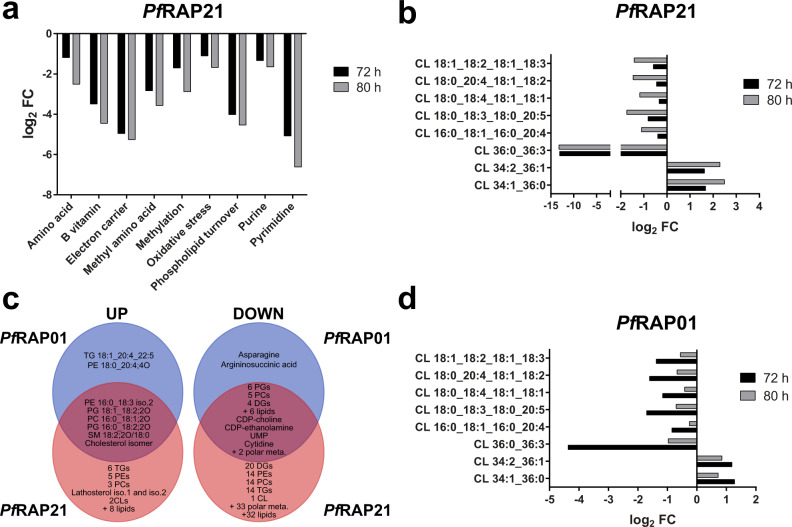
Metabolomics analyses of *Pf*RAP01 and *Pf*RAP21 knockdowns. **a** Average relative log2 FC (−aTc/+aTc) of the polar metabolites grouped in their respective pathways from *Pf*RAP21 samples at 72 and 80 h. **b** Relative log2 FC (−aTc/+aTc) of cardiolipins (CL) detected in lipidomics analysis of *Pf*RAP21 lines. **c** Distribution of metabolites significantly affected in *Pf*RAP01 and *Pf*RAP21 lines. The total number of metabolites in each subgroup is indicated in red. **d** Relative log2 FC (−aTc/+aTc) of cardiolipins (CL) detected in lipidomics analysis of *Pf*RAP01 lines.

To better understand the metabolic pathways affected by protein knockdown, we performed targeted metabolomics and untargeted lipidomics analyses to assess the effects of *Pf*RAP01 and *Pf*RAP21 loss. Synchronized rings were cultured in the presence or absence of aTc and triplicate samples were collected at 56, 72, and 80 h in the second IDC (Fig. 4a), corresponding to just before and during the death phenotype. We successfully detected 120 and 93 polar metabolites, and 558 and 511 lipids in the *Pf*RAP01 and *Pf*RAP21 samples, respectively (Supplementary Data 3). No major changes in ring stage were detected with only one and four metabolites/lipids significantly impacted in *Pf*RAP01 and *Pf*RAP21. For *Pf*RAP21, 32 lipids showed higher abundance levels in deficient parasites at 72 and 80 h. These lipids correspond, among others, to 6 phosphatidylethanolamines (PEs), 6 triglycerides (TGs), 2 cardiolipins, and 3 sterols (Supplementary Data 3). As *P. falciparum* cannot synthesize sterols and scavenge them from the host, this upregulation is probably unrelated to the function of RAP proteins and due to the growth defect. Conversely, we identified a significant decrease in the relative abundance of 39 polar metabolites and 116 unique lipids, which could arise due to cell cycle arrest upon *Pf*RAP21 knockdown. Mainly, these metabolites were composed of 24 diglycerides, 19 phosphatidylcholines (PCs), 16 TGs, 14 PEs, and 13 phosphatidylglycerol (PGs) (Supplementary Data 3). The lower abundance of CDP-ethanolamine, CDP-choline, and P-choline confirmed this global down-regulation of the phospholipid turnover (Fig. 5a and Supplementary Fig. 4b). Although inhibition of the mitochondrial electron transport chain has been shown to affect choline and PCs abundance in neuroblastoma cells^33^, it is likely that this effect in our studies is a consequence of parasite death. However, the two most impacted pathways correspond to electron carriers (FAD and NAD) and polar metabolites involved in pyrimidine biosynthesis (Fig. 5a and Supplementary Fig. 4b), both of which being required for and/or dependent on mitochondrial functions. Detailed analysis also showed alteration in the abundance of 8 cardiolipins, which are essential constituents of mitochondrial membranes and involved in various mitochondrial processes. Two were significantly more abundant and one was not detected in the *Pf*RAP21 -aTc samples, while the remaining five cardiolipins were only significantly impacted at 80 h (Fig. 5b). Altogether, these results suggest that the disruption of *Pf*RAP21 affects overall metabolic activity with a higher impact on mitochondrion-dependent pathways. In the *Pf*RAP01 knockdown parasites, we observed a significant increase in the relative abundance of 8 lipids (2 PEs, 2 PGs, 1 PC, 1 TG), and decrease of 8 polar metabolites and 21 lipids (Supplementary Data 3 and Supplementary Fig. 4a). Interestingly, 33 of these affected metabolites were similarly affected in *Pf*RAP21 (Fig. 5c), confirming the same trend despite the lower efficiency of the knockdown system.

### Impact on mitochondrial electron transport in *Pf*RAP01 and *Pf*RAP21 knockdown parasites

To evaluate whether a defect in mitochondrial membrane potential (Δψ_m_) occurred in *Pf*RAP01 and *Pf*RAP21 knockdown parasites, we performed live-cell imaging with MitoTracker staining. Mitochondrial structure was observed for *Pf*RAP01 and *Pf*RAP21 lines ± aTc at 72 and 80 h (Supplementary Fig. 5a–c). A higher number of tubular mitochondria was detected for parasites expressing *Pf*RAP01 or *Pf*RAP21 at both time points. However, this difference is likely due to the cell cycle arrest previously described. Indeed, mitochondrial morphology evolves during parasite maturation and tubular structures are observed in late asexual stages, as confirming by the higher number detected at 80 h compared to 72 h. No diffuse staining was observed as described previously with the knockdown of the mitochondrial ribosomal protein L13 (PfmRPL13)^34^.

To further explore the impact on mitochondrial metabolism, we carried out a differential sensitivity assay by treating the conditional knockdown lines with atovaquone, an inhibitor of the mitochondrial electron transport required to sustain de novo pyrimidine biosynthesis. We first determined the IC50 values obtained with aTc for *Pf*RAP01 and *Pf*RAP21 lines. For both proteins, we observed a steep dose-response curve suggesting an “all or nothing” action of the aTc with IC50 at 8 and 5 nM for *Pf*RAP01 and *Pf*RAP21, respectively (Supplementary Fig. 5d). We then performed drug inhibition assays beginning with ring-stage parasites with high (500 nM), low (IC values), or in absence of aTc. Analysis of atovaquone dose-response curves revealed no change in sensitivity of either *Pf*RAP01 or *Pf*RAP21 in high and low aTc conditions (Supplementary Fig. 5e, f), similar to an aptamer-regulated YFP control line (Supplementary Data 4). This absence of hypersensitivity could be due to sufficient expression of each RAP protein at these concentrations of aTc. However, parasites were too affected without aTc to assess the impact of atovaquone. Additionally, with two control compounds that do not directly target mitochondrial functions, quinine and Bafilomycin A1 (a V-type ATPase inhibitor), no change in parasite sensitivity was observed upon *Pf*RAP01 or *Pf*RAP21 knockdown. Thus, conditional depletion of *Pf*RAP01 and *Pf*RAP21 does not appear to increase parasite sensitivity to atovaquone. Knockdown of the mitochondrial PfmtRPS17 protein, with a close phenotype, showed hypersensitivity in a nonspecific manner indicating a minor roles of mitochondrial drugs on severe knockdowns^35^.

### Identification of RAP-protein complexes

To examine further the functions of our RAP proteins, we sought to determine their potential protein and RNA-binding partners. We performed anti-HA immunoprecipitation (IP) and mass spectrometry analysis on soluble protein fractions extracted from the *Pf*RAP01-HA, *Pf*RAP21-HA, and the parental lines (negative control) (Supplementary Data 5). Proteins were filtered with QSPEC-calculated Log FC ≥ 1 and *p* value ≤0.05 compared to values measured from the parental line. With an average dNSAF value of 0.21, *Pf*RAP01 was by far the most abundant protein in its HA-affinity purification (Supplementary Fig. 6a). One other protein, PF3D7_0106300 ATP6, a calcium-transporting ATPase, was detected at a much lower level (about 100 times less). As ATP6 is not predicted to be a mitochondrial protein based on its sequence analysis by MitoProt II (Supplementary Data 5), it is unlikely to be a viable interacting partner. While the *Pf*RAP21 bait was detected at lower levels (its spectral counts contributed <1% of the total spectra in its HA purification), three proteins (PF3D7_0703500, PF3D7_1237100, and PF3D7_0924200) were reproducibly and significantly enriched in the replicate *Pf*RAP21 IP samples (Supplementary Fig. 6b). All three proteins were recovered at abundance levels similar to *Pf*RAP21 and are predicted to localize to the mitochondrion. While annotated as an erythrocyte membrane-associated antigen, PF3D7_0703500 has one N-terminal membrane anchor and a DEAD-like helicase superfamily domain suggesting this protein could be involved in protein transport and/or mRNA-binding. PF3D7_1237100 is also predicted to contain several transmembrane domains. For PF3D7_0924200, the protein exhibits a heptatricopeptide-repeat domain, most likely interacting with RNA^20^. Although these findings will require further validation, they suggest that *Pf*RAP01 may operate independently while *Pf*RAP21 might be part of a complex to perform their critical mitochondrial functions.

### Identification of RAP-RNA complexes

**Fig. 6:**
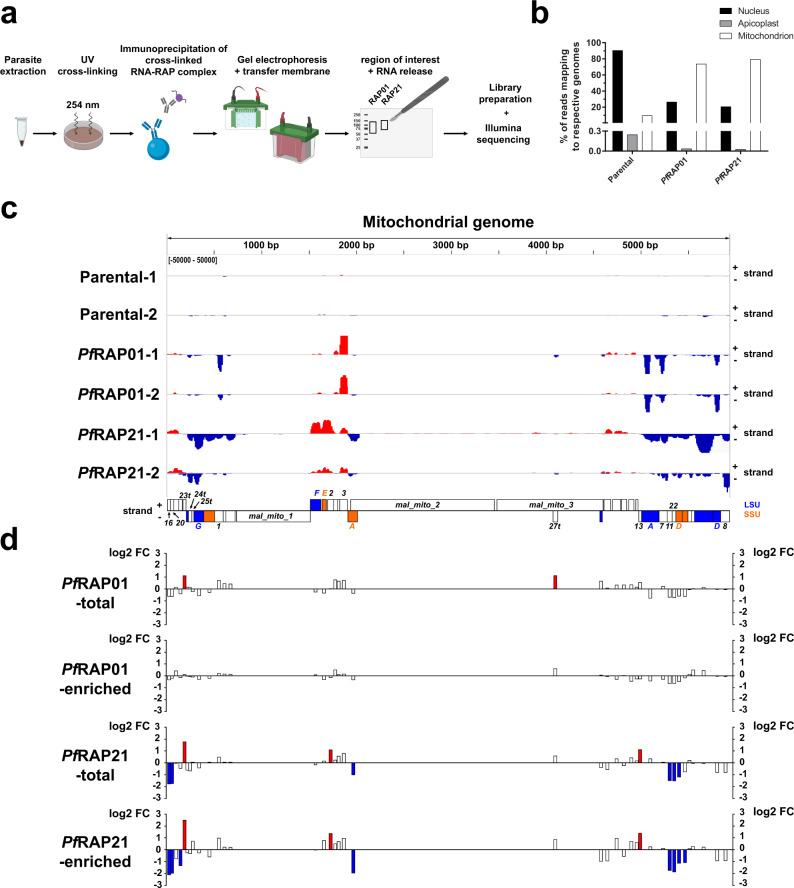
*Pf*RAP01 and *Pf*RAP21 participate in mitoribosome regulation. **a** The schematic illustrates the key steps of the eCLIP-seq protocol. Created with BioRender.com. **b** Distribution of the reads to the nuclear and mitochondrial genome. **c** Read density tracks along the mitochondrial genome for parental, *Pf*RAP01, and *Pf*RAP21 samples (*n* = 2). SSU = small subunit (orange), LSU = large subunit (blue). **d** Log2 FC values determined for mitochondrial rRNAs from total and enriched RNA of *Pf*RAP01 and *Pf*RAP21 lines. rRNAs identified as differentially up-regulated and down-regulated (log2 FC > 1 or <1) are respectively indicated in red and blue.

To identify potential RNAs directly interacting with *Pf*RAP01 and *Pf*RAP21 proteins, we performed the first eCLIP-seq experiment in *P. falciparum*. Briefly, RNA-protein complexes from the parental, HA-tagged *Pf*RAP01 and *Pf*RAP21 lines, were UV-crosslinked and immunoprecipitated (Fig. 6a). After gel electrophoresis and transfer onto nitrocellulose membrane, RNAs were released by proteinase K treatment for library preparation and high-throughput sequencing on the Illumina NOVAseq platform. eCLIP-seq experiments were performed in duplicate. Approximately 76% of the reads from the *Pf*RAP01 and *Pf*RAP21 lines mapped to the mitochondrial genome, compared to 10% from the parental control, validating significant enrichment of mitochondrial reads in all RAP samples (Fig. 6b). Using the MACS2 peak caller, we observed that the non-mitochondrial peaks detected were mainly related to rRNAs, tRNAs, and snRNAs (Supplementary Data 6). However, analysis of normalized read counts indicated that most of these genes were also covered in the parental samples (Supplementary Fig. 7a). MACS2 detected the entire mitochondrial genome with the highest −log10 (*q* value) in both RAP samples (x2.8 higher than the 2nd highest peak) (Supplementary Data 6). Detailed analysis of normalized read counts showed that although the entire mitochondrial genome was called, *Pf*RAP01 and *Pf*RAP21 interacted mainly and specifically with rRNAs (Fig. 6c). *Pf*RAP01 mainly interacted with rRNA1, rRNA3, rRNA7, and large subunit ribosomal RNA (LSU) fragments A and D, while *Pf*RAP21 bound to rRNA3, rRNA7, rRNA8, rRNA24t, rRNA25t, small subunit ribosomal RNA (SSU) fragment E, and LSU fragments F and G. These rRNAs are associated with both SSU and LSU rRNAs^35,36^, known to form the mitoribosome, suggesting that binding is not subunit-specific. Likewise, the orientation of the reads showed a nearly perfect correlation with the positioning of the genes on the plus and minus strands (Fig. 6c), validating that the RAP proteins interacted directly with the rRNAs and not the intergenic regions or opposite strand of the mitochondrial genome. These rRNAs are completely covered, with the exception of rRNA1, where *Pf*RAP01 reads map mainly to the 5′ side, and rRNA7 and LSU fragments A and D where *Pf*RAP01 reads map to the 3′ end. No significant RNA motifs representing potential *Pf*RAP01 or *Pf*RAP21 binding sites could be identified using the MEME suite^37^. Loss of *Pf*RAP01 and *Pf*RAP21 proteins could potentially result in mitoribosome destabilization, leading to alteration of mitochondrial functions and initiate a cascade of events lethal to the parasite. Altogether, the eCLIP-seq data confirmed that *Pf*RAP01 and *Pf*RAP21 are true RBPs, and allowed identification of their RNA targets and their importance in mitoribosome function.

### Dysregulation of mitoribosome expression in *Pf*RAP01 and *Pf*RAP21 knockdown parasites

To investigate the impact of *Pf*RAP01 and *Pf*RAP21 knockdowns on mitochondrial rRNAs, we performed small RNA library preparation from total parasites and from organelles isolated by N_2_ cavitation (=enriched RNA). The samples were prepared in duplicate at 72 h after aTc removal and the libraries were size-selected for cDNA fragments between 140 and 350 nucleotides before sequencing (Supplementary Fig. 8a). Approximately 40% of the reads from the enriched RNA mapped to the mitochondrial genome, compared to 12% from the total RNA, validating significant enrichment of mitochondrial reads (Supplementary Fig. 8b, c). In all samples, high numbers of reads were mapped to rRNAs and tRNAs (32–59% of total reads) (Supplementary Fig. 8d). Among the 45 nuclear tRNAs, 25% were up-regulated (log2 FC > 1) in both RNA preparations in *Pf*RAP21-deficient parasites (Supplementary Data 7), confirming the increase observed for the genes involved in translation in our RNA-seq data. The levels of rRNA expression were similar for the two different RNA isolation methods, validating the results obtained. As expected, mitochondrial reads mapped predominantly to rRNAs. Upon knockdown of *Pf*RAP21, 6 rRNAs were down-regulated (rRNA16, rRNA20, rRNA11, rRNA22, and SSU fragments A and D) while rRNA23t, rRNA2 rRNA13 were up-regulated (Fig. 6d). The majority of these rRNAs showed a similar trend in *Pf*RAP01-deficient parasites, although only rRNA23t and rRNA27t showed differential expression in total RNA samples (Fig. 6d). This low impact can be explained by the incomplete down-regulation of *Pf*RAP01 as previously noticed (Fig. 3a). The results suggest that the level of these ribosomal subunits can be controlled by each RAP protein (Supplementary Fig. 8e). It is however important to note that the most affected rRNAs did not perfectly match the strongest eCLIP-seq binding peaks, which appear to be positioned upstream. This result suggests that the binding of RAP proteins upstream of targeted rRNAs might be necessary to fulfill their function, although this hypothesis will need to be further investigated. Despite the low coverage of nuclear genes, only one rRNA (PF3D7_0112500) was significantly impacted in both conditions in *Pf*RAP21 samples indicating that the RAP protein knockdowns had almost no impact on nuclear ribosomes (Supplementary Data 7). The knockdowns of *Pf*RAP01 and *Pf*RAP21 thus showed an imbalance in the mitochondrial rRNAs, which could prompt a defect in the mitoribosome function.

## Discussion

Mitochondrial genomes in apicomplexan parasites present atypical features, with only three protein-coding genes and multiple highly fragmented rRNAs^36,38,39^. This strong reduction of the genome size requires import of nuclear-encoded proteins to ensure the various essential functions of this organelle^40–42^. In *P. falciparum*, which has one of the smallest mitochondrial genomes^43,44^, hundreds of proteins are imported through a system requiring transit peptides and import machinery involving TOM and TIM proteins^45,46^. Understanding the exact function of parasite specific proteins that are imported to the mitochondria could lead to discovery of new therapeutic strategies. Here, we characterized two RAP proteins encoded by the nuclear genome, but localized to the parasite mitochondrion. Their localization is consistent with the protein family, whose members are associated with mitochondria or plastids in various organisms^7–12^. IFA studies indicated that *Pf*RAP01 and *Pf*RAP21 are expressed in late asexual stages, consistent with Western blot analysis and previous transcriptomic data^27–29^. Using CRISPR/Cas9 genome editing, we generated two inducible knockdown transgenic lines and showed that both RAP proteins are essential for the asexual blood stages, validating previous large-scale screening data in *P. falciparum* and *P. berghei*^22,23^. Parasite growth arrest was observed 72–80 h after aTc removal in the trophozoite stage of the subsequent IDC. The latency period seems to be due to significant loss of RAP proteins, which is only achieved during the second IDC, as confirmed by the growth after replenishment at 56 h. A similar delayed response was also observed with conditional knockdown of PfmtRPS17, a mitochondrial ribosomal protein^35^. An even higher number of cycles was necessary to observe parasite growth defect after knockdown of two other mitochondrial proteins, PfmRPL13^34^ and PfmtRPS12^35^.

Transcriptomics analysis performed to determine the pathways impacted in the knockdown parasites led to an arrest in cell cycle and overexpression of genes implicated in RNA biology, confirming the predicted involvement of the RAP proteins in this particular pathway. Several other RAP proteins were also significantly up-regulated, potentially to partially compensate for lack of *Pf*RAP01 and *Pf*RAP21. Although not statistically significant, the three mitochondrial protein-coding genes were down-regulated, suggesting potential involvement of *Pf*RAP01 and *Pf*RAP21 proteins in the regulation of their expression.

Although it is difficult to distinguish direct and indirect effects of protein knockdown by metabolomics profiling, our results indicate that *Pf*RAP21 is most likely associated with mitochondrial dysfunction since knockdown led to a decrease in the levels of electron carriers and polar metabolites involved in pyrimidine biosynthesis. Although few metabolites were detected as significantly altered by *Pf*RAP01 knockdown, we noticed a trend wherein levels of the same compounds were perturbed in both lines. Metabolic profiling of PfmtRPS17 showed a decrease in abundance of ten metabolites associated with the pyrimidine de novo synthesis pathway in the absence of aTc^35^, consistent with our results. Only two intermediates, N-carbamoyl-l-aspartate and dihydroorotate, accumulated in the deficient parasites, but these were not detected in this study.

Although destabilization of mitochondrial electron transport was not detected upon *Pf*RAP01 and *Pf*RAP21 knockdown, an effect on this pathway cannot be excluded. In previous studies, hypersensitivity to the mitochondrial drugs (atovaquone, DSM265, and proguanil) was detected with PfRPS12^35^ and PfmRPL13^34^, two mitochondrial proteins whose knockdown caused a medium loss of fitness with effects on parasite growth occurring only 6–8 days after aTc removal. PfmtRPS17, with a rapid onset phenotype similar to *Pf*RAP01 and *Pf*RAP21, was hypersensitive to all inhibitors in a nonspecific manner confirming the difficulty to detect this synergy with severe knockdowns^35^.

IP-MS experiments identified three candidate partners (PF3D7_0703500, PF3D7_1237100, and PF3D7_0924200) of *Pf*RAP21. Although limited information is available on these proteins, they are predicted to be localized to the mitochondrion and potentially associated with RNA metabolism. Additional experiments will be necessary to validate these partners and exclude the possibility of false-positive interactions or indirect association through RNA bridging. However, it is interesting to note that PF3D7_0924200 is a HPR protein and HPR domains have also been identified in other RAP proteins, including *Pf*RAP01, and could participate in RNA binding^20^. Although several RAP proteins do not have detectable HPR or PPR motifs, the N-terminal helical structure regions are conserved between all RAP proteins^7^. Further studies will be required to assess the contribution of these regions to RNA binding, and to understand what are the critical interactions for regulating proper mitoribosome function.

Adaptation of the eCLIP-seq methodology to *Plasmodium* allowed us to identify and characterize the RNA targets of *Pf*RAP01 and *Pf*RAP21 proteins in situ. We successfully demonstrated that each protein binds to distinct mitochondrial rRNA transcripts associated with the small subunit (SSU) and large subunit (LSU) rRNAs, which are thought to form the mitoribosome. To further validate our eCLIP-seq results, we sequenced small RNAs from total and mitochondria-enriched RNA samples. While only a few rRNAs were significantly affected by *Pf*RAP01 knockdown, 9 mitochondrial rRNAs were significantly up- and down-regulated for *Pf*RAP21. This difference could arise due to the more efficient depletion of *Pf*RAP21 compared to *Pf*RAP01. The discrepancies observed between the eCLIP-seq and the small RNA-seq results might indicate that the RAP proteins recognize upstream regions of the targeted rRNAs. It is also possible that these proteins interact on polycistronic precursor transcripts prior to maturation events, leading to the high number of binding sites identified by eCLIP-seq. However, a role during the assembly of mitoribosome can also not be excluded. Identifying these binding sites could clarify how and why each protein recognizes and targets these rRNAs. Overall, eCLIP-seq and small RNA-seq validated the involvement of *Pf*RAP01 and *Pf*RAP21 in regulation of the parasite mitoribosome.

In Apicomplexa, the mitochondrial genome exhibits atypically high fragmentation, with 39 rRNAs and only three protein-coding genes^36,38,39^. This contrasts with the human mitochondrial genome, which has 2 rRNAs and 13 genes encoding the protein subunits of respiratory complexes. The considerable expansion of the number of RAP proteins in apicomplexan parasites may reflect a requirement for supplemental proteins to counteract the rRNA fragmentation and to allow assembly of a functional mitoribosome. Some alveolates such as *Tetrahymena thermophila* and *Symbiodinium microadriaticum*, or green alga (e.g., *Gonium pectorale* or *Monoraphidium neglectum*) corroborate this interpretation since their mitochondrial genome is fragmented to a lesser degree and have an intermediate number of RAP proteins^7^. The deficiency in *Pf*RAP01 and *Pf*RAP21 could hamper the stability of fragmented rRNAs and/or mitoribosome assembly leading to down-regulation of the three mitochondrial protein-coding genes as detected in our RNA-seq. This could destabilize mitochondrial respiration and associated metabolic pathways leading ultimately to parasite death. Altogether our results confirm an essential role of *Pf*RAP01 and *Pf*RAP21 proteins in regulation of the parasite’s mitoribosome. Although the RAP domain is evolutionarily conserved^7^, the high variability in the N-terminal regions of these proteins, especially with human FASTKs, could open new opportunities for selectively targeting the *Plasmodium* proteins for therapeutic purposes.

## Methods

### Transfections and cultures of *P. falciparum*

The *P. falciparum* lines were grown as previously described^47^ in 5% of human O + erythrocytes. Parasites were synchronized by two D-sorbitol treatments^48^ and liberated from red blood cells with 0.15% saponin^49^.

Using CRISPR/Cas9, we generated parasite lines where we fused the *pf3d7_0105200* and *pf3d7_1470600* transcripts with a C-terminal 3x-HA epitope tag and RNA aptamers for translational regulation with the TetR-DOZI module^25,26^. To construct the modification plasmids, we cloned the target specifying sequences, the right homology regions, and the left homology regions (LHR) of each gene into the pSN053 linear vector^25^ via Gibson assembly. The homology arms were PCR amplified and gBlock synthesized (IDT-DNA) recodonized 3′-end of each target genes were fused to the LHR to prevent cleavage of the modified locus. The target specifying guide RNA sequences were generated by Klenow reaction. In addition to the gene expression regulation module, the pSN053 vector also features the reporter construct *Renilla luciferase* (*Rluc*) and the selection marker *blasticidin S-deaminase* gene. The final donor vectors were confirmed by restriction digests and Sanger sequencing. Primers used in this study are listed in Supplementary Data 8.

Transfections into Cas9- and T7 RNA polymerase-expressing NF54 parasites were carried out by preloading erythrocytes with the donor plasmids as described previously^50^. Cultures were maintained in 500 nM anhydrotetracycline (Sigma-Aldrich, 37919) and 2.5 µg/ml of Blasticidin (RPI Corp B12150-0.1). Parasite cell lines stably integrating the donor plasmids were monitored via Giemsa smears and Rluc measurements using the Renilla-Glo^®^ Luciferase Assay System (Promega E2750) and the GloMax® Discover Multimode Microplate Reader (Promega).

### Transgenic lines validation

Parental and inducible lines were cloned by limiting dilution. Infected red blood cells were lysed with DNeasy Blood & Tissue kit (Qiagen) to extract genomic DNA. Primers used to genotype *pf3d7_0105200-HA* and *pf3d7_1470600-HA* are listed in Supplementary Data 8.

For whole-genome sequencing, genomic DNAs were fragmented using Covaris ultrasonicator and libraries were generated using KAPA LTP Library Preparation Kit (Roche, KK8230). Edited genomes were constructed by adding the desired inserted sequences into the *P. falciparum* genome, version 43 (http://plasmodb.org). The reads were aligned to these edited genomes using Bowtie2 with default settings^51^. Reads that aligned with mapping quality of 40 or below were filtered out using Samtools^52^ (Supplementary Data 9). Alignments were visualized on IGV^53^. Sequence reads have been deposited in the NCBI Sequence Read Archive with accession number PRJNA690830.

The expression of *Pf*RAP01 and *Pf*RAP21 across the asexual cycle and at trophozoite stages (24 and 72 h after aTc removal) was assessed by Western blot. Blots were probed with anti-HA antibody (1:2500, Abcam, ab9110) and anti-*Plasmodium* aldolase (1:10000, Abcam, ab207494) followed by HRP-labeled Goat anti-Rabbit IgG (H + L) (1:10,000, Novex^TM^, A16104). Chemiluminescence detection with Clarity^TM^ Western ECL Substrate (Bio-Rad, 1705060) was applied to reveal the blots. Relative abundance of the RAP proteins was normalized with *Pf*-aldolase expression using Image Lab software (Bio-Rad).

### Immunofluorescence assays

*Pf*RAP01-HA and *Pf*RAP21-HA parasites ± aTc were washed in incomplete medium then incubated with 0.5 µM of MitoTracker^TM^ Red CM-H_2_Xros (Invitrogen, M7513) for 30 min at 37 °C. Parasites were washed in incomplete medium and fixed with 4% paraformaldehyde and 0.0075% glutaraldehyde for 15 min at 4 °C, then sedimented on Poly-D-lysine coated coverslips for 1 h at room temperature. After PBS washing, cells were permeabilized and saturated with 0.2% Triton X-100, 5% BSA, 0.1% Tween 20 in PBS for 30 min at room temperature. Anti-HA mAb (Abcam, ab24779) was diluted at 1:500 in 5% BSA, 0.1% Tween 20 and PBS, and applied for 1 h at room temperature, followed by Goat anti-Mouse Alexa Fluor 488 (1:2000, Invitrogen, A11001) for 1 h at room temperature. The coverslips were mounted in Vectashield Antifade Mounting Medium with DAPI (H-1200). The rabbit anti-Cpn60 (kindly provided by Dr. Boris Striepen) was used in the same condition at 1:1000 with Donkey anti-Rabbit Alexa Fluor 568 (1:2000, Invitrogen, A10042). Images were acquired using Zeiss LSM880 microscope with Airyscan (Fig. 2) or Leica DMI 6000 (Supplementary Figs. 2 and 3) and treated with ImageJ. Co-localizations of *Pf*RAP01 and *Pf*RAP21-HA signals with MitoTracker or Cpn60 were quantified on trophozoite stage by measuring the Pearson correlation coefficient ± SD using JACoP (*n* = 10).

### Parasitemia and phenotype analyses

To determine the essentiality of the RAP proteins, we synchronized Parental F3, *Pf*RAP01 F2 and F4, and *Pf*RAP21 G4 and G1 lines. Parasitemia and proportion of the different asexual blood stages were determined by counting Giemsa-stained blood smears, under the microscope at the indicated time points (*n* = 51–106 parasites counted in duplicate). For aTc replenishment, synchronous rings were cultured with or without aTc. At 32, 56, or 72 h, parasites were replenished with 500 nM of aTc. The viability of the cultures was assessed by DNA quantification using SYBR Green (Thermo Fisher, S7523) at 80 and 128 h.

### RNA-sequencing

Parasites in ring, trophozoite, and schizont stages (5 × 10^8^ cells) were extracted by saponin treatment before flash freezing. Two independent biological replicates were generated for each time point, culture condition, and line. Total RNA was extracted with TRIzol*®* LS Reagent (Invitrogen, 10296028) then incubated for 1 h at 37 °C with 4 units of DNase I (NEB, M0303). RNA samples were visualized by RNA electrophoresis and quantified on Synergy^TM^ HT (BioTek). Then mRNAs were purified using NEBNext® Poly(A) mRNA Magnetic Isolation Module (NEB, E7490) according to the manufacturer’s instructions. Libraries were prepared using NEBNext® Ultra^TM^ Directional RNA Library Prep Kit (NEB, E7420L). Final libraries were amplified by PCR with KAPA HiFi HotStart Ready Mix (KAPA Biosystems, KK2602) and the PCR conditions consisted of 15 min at 37 °C followed by 12 cycles of [98 °C (30 s), 55 °C (10 s) and 62 °C (1 min 15)], finished by 5 min at 62 °C. The quantity and quality of the final libraries were assessed using a Bioanalyzer (Agilent Technology Inc). All samples were multiplexed and sequenced on 100 nucleotides paired-end run on the Illumina NovaSeq 6000 sequencer at the UC San Diego IGM Genomics Center to produce at least 10 million of reads per sample (Supplementary Data 9). FastQC^54^ was used to assess raw read quality. Adapter sequences as well as the first 11 bp of each read were removed using Trimmomatic^55^. Tails of reads were trimmed using Sickle^56^ with a Phred base quality threshold of 20, and reads shorter than 18 bp were removed. To remove human RNA contamination, reads were then aligned against the *H. sapiens* genome (assembly GRCh38) using Bowtie2 (version 2.3.4.1)^51^, and unmapped reads were retained. These reads were then aligned to the *P. falciparum* genome (version 43, http://plasmodb.org) using HISAT2^57^. Only properly paired reads with a mapping quality score of 40 or higher were retained, with filtering done using Samtools^52^. Raw read counts were determined for each gene in the *P. falciparum* genome using BedTools^58^ multicov to intersect the aligned reads with the genome annotation. DESeq2^59^ was then used for differential expression analysis. Only the genes with log2 FC > 1 or <1 and adjusted *p* value <0.05 at 72 and 80 h were included in heatmaps. R package pheatmap^60^ was used to generate heatmaps. PlasmoDB was used for GO enrichment analysis. Sequence reads have been deposited in the NCBI Sequence Read Archive with accession number PRJNA690830.

### Metabolomics sample preparation

Tightly synchronized parasites (5 × 10^9^ parasites at 56 h and 9 × 10^8^ parasites at 72 and 80 h), were lysed with saponin, flash frozen and stored at −80 °C. Lipids and polar metabolites were extracted from malaria pellets using a biphasic approach. To each sample, 1 ml of ice cold 3:2 methyl tert-butyl ether:80% methanol was added. To break up malaria pellets, samples were vortexed 2 min, sonicated for 15 min, vortexed for 2 min, sonicated for 15 min, then vortexed for 30 min at 4 °C. All sonication was performed in an ice bath. In total, 200 µl of water was added to induce phase separation, followed by a 5 min vortex. After centrifugation for 15 min at 4 °C at 16,000 × *g*, 200 µl of the top, nonpolar layer was transferred to a 2 ml glass vial and the bottom, polar layer was transferred to a new 2 ml glass vial then analyzed by LC-MS. The nonpolar fraction was dried under a gentle stream of nitrogen at room temperature then resuspended in 400 µl of 9:1 methanol:toluene and analyzed by LC-MS.

### LC-MS lipidomics

LC-MS lipidomics analysis was performed at the UC Riverside Metabolomics Core Facility as described previously^61^, with minor modifications. Briefly, analysis was performed on a Waters G2-XS quadrupole time-of-flight mass spectrometer coupled to a Waters Acquity I-class UPLC system. Separations were carried out on a Waters CSH C18 column (2.1 × 100 mm, 1.7 µM). The mobile phases were (A) 60:40 acetonitrile:water with 10 mM ammonium formate and 0.1% formic acid and (B) 90:10 isopropanol:acetonitrile with 10 mM ammonium formate and 0.1% formic acid. The flow rate was 400 µl/min and the column was held at 65 °C. The injection volume was 2 µl. The gradient was as follows: 0 min, 10% B; 1 min, 10% B; 3 min, 20% B; 5 min, 40% B; 16 min, 80% B; 18 min, 99% B; 20 min 99% B; 20.5 min, 10% B. The MS was scan range was (50–1600 *m*/*z*) with a 100 ms scan time. MS/MS was acquired in data dependent fashion. Source and desolvation temperatures were 150 °C and 600 °C, respectively. Desolvation gas was set to 1100 l/h and cone gas to 150 l/h. All gases were nitrogen except the collision gas, which was argon. Capillary voltage was 1 kV in positive ion mode. A quality control sample, generated by pooling equal aliquots of each sample, was analyzed periodically to monitor system stability and performance. Samples were analyzed in random order. Leucine enkephalin was infused and used for mass correction.

### LC-MS metabolomics—polar metabolites

Targeted metabolomics of polar, primary metabolites was performed on a TQ-XS triple quadrupole mass spectrometer (Waters) coupled to an I-class UPLC system (Waters). Separations were carried out on a ZIC-pHILIC column (2.1 × 150 mm, 5 µM) (EMD Millipore, 150460). The mobile phases were (A) water with 15 mM ammonium bicarbonate adjusted to pH 9.6 with ammonium hydroxide and (B) acetonitrile. The flow rate was 200 µl/min and the column was held at 50 °C. The injection volume was 2 µl. The gradient was as follows for *Pf*RAP01: 0 min, 90% B; 1.5 min, 90% B; 16 min, 20% B; 18 min, 20% B; 20 min, 90% B; 28 min, 90% B, and as follows for *Pf*RAP21: 0 min, 90% B; 1.5 min, 90% B; 16 min, 10% B; 18 min, 10% B; 20 min, 90% B; 28 min, 90% B.

The MS was operated in selected reaction monitoring mode^62^. Source and desolvation temperatures were 150 °C and 600 °C respectively. Desolvation gas was set to 1100 l/h and cone gas to 150 l/h. Collision gas was set to 0.15 ml/min. All gases were nitrogen except the collision gas, which was argon. Capillary voltage was 1 kV in positive ion mode and 2 kV in negative ion mode. System stability was monitored by analyzing a quality control sample (generated by pooling together equal volumes of all sample extracts) every 3 injections. Samples were analyzed in random order.

### Data processing and analysis

Targeted data processing (manual peak integration) was performed in Skyline software^63^. Untargeted data processing (peak picking, alignment, deconvolution, integration, normalization, and spectral matching) was performed in Progenesis Qi software (Nonlinear Dynamics). Metabolomics data were normalized by parasite count and lipidomics data were normalized to total ion abundance. Lipidomics features with a CV greater than 30% across QC injections were removed^64,65^. To aid in the identification of features that belong to the same metabolite, features were assigned a cluster ID using RAMClust^66^. An extension of the metabolomics standard initiative guidelines was used to assign annotation level confidence^67,68^. Annotation level 2a indicates an MS and MS/MS match to an external database. Level 2b indicates an MS and MS/MS match to the Lipidblast in silico database^69^ or an MS match and diagnostic evidence. Several mass spectral metabolite libraries were searched including those in Mass Bank of North America, Metlin^70^, and an in-house library.

Metabolites were described as significantly affected if *p* value <0.05 and log2 FC < −1 or >1 at 56 h or both at 72 and 80 h.

### Live-cell imaging

*Pf*RAP01 and *Pf*RAP21 knockdown lines were washed in incomplete medium then incubated with 0.5 µM of MitoTracker^TM^ Red CM-H2Xros (Invitrogen, M7513) and 8 µM Hoechst 33342 (Invitrogen, H3570) for 30 min at 37 °C. Parasites were washed in incomplete medium and were mounted between slide and coverslip. Images were acquired using Leica DMI 6000 and treated with ImageJ. At least 30 parasites were used for the quantification.

### Compound susceptibility assays

Serial dilutions of aTc, atovaquone, quinine, and bafilomycin A were generated to yield final concentrations ranging from 40–0.462 nM, 25–0.048 nM, 432–0.84 nM, and 40–0.08 nM, respectively. Synchronous ring-stage *Pf*RAP01 and *Pf*RAP21 conditional knockdown lines as well as a control cell line expressing an aptamer-regulatable fluorescent protein were maintained in high aTc (500 nM), low aTc in the case of *Pf*RAP01 (8 nM) and *Pf*RAP21 (5 nM) or no aTc, and were distributed into 384-well assay plates (Corning^®^, 89176-442). Compounds were transferred to the parasite-containing plates using the Janus^®^ platform (PerkinElmer). DMSO- and dihydroartemisinin-treatment (500 nM) served as reference controls. Growth inhibition was analyzed after 72 and 120 h using the Renilla-Glo(R) Luciferase Assay System (Promega, E2750) and the GloMax® Discover Multimode Microplate Reader (Promega). IC_50_ values were obtained from corrected dose-response curves and plotted using GraphPad Prism 8.

### Immunoprecipitation followed by MudPIT mass spectrometry

Purified late asexual parasites from parental, *Pf*RAP01, and *Pf*RAP21 lines (7.5 × 10^9^ to 1.5 × 10^10^ cells) were suspended in 50 mM Tris-HCl pH 7.5, 150 mM NaCl, 5 mM EDTA, 1 % Triton X-100, 1 mM AEBSF and EDTA-free protease inhibitor cocktail (Roche, 11873580001). After lysis by passing 25 times through a 26G needle, the soluble extracts were treated with 100 units of DNase I (NEB, M0303) for 10 min at room temperature and centrifuged at 14,000 × *g* for 15 min at 4 °C. The lysates were precleared with Dynabeads^TM^ Protein A (Invitrogen, 10001D) for 1 h at 4 °C. The Rb anti-HA antibody (1:100, Abcam, ab9110) was added in each sample and incubated overnight at 4 °C. Dynabeads^TM^ Protein A were used to precipitate antibody-protein complexes and were washed with buffer A (1 % Triton X-100, 1 mM EDTA in PBS), buffer B (wash buffer A, 0.5 M NaCl) and buffer C (1 mM EDTA in PBS). Proteins were eluted using 0.1 M glycine, pH 2.8, and neutralized using 2 M Tris-HCl, pH 8.0. Then proteins were precipitated in 20% TCA followed by cold acetone washes.

TCA-precipitated proteins were urea-denatured, reduced, alkylated, and digested with endoproteinase Lys-C (Promega, V1671) followed by modified trypsin (Promega, V5111)^71^. Peptide mixtures were loaded onto 100 μm fused silica microcapillary columns packed with 5-μm C18 reverse phase (Aqua, Phenomenex), strong cation exchange resin (Luna, Phenomenex), and 5-μm C18 Aqua^71^. Loaded microcapillary columns were placed in-line with a Quaternary Agilent 1100 series HPLC pump and a LTQ linear ion trap mass spectrometer equipped with a nano-LC electrospray ionization source (Thermo Scientific, San Jose, CA). Fully automated 10-step MudPIT runs were carried out on the electrosprayed peptides, as previously described^71^. Tandem mass (MS/MS) spectra were interpreted using ProluCID^72^ v.1.3.3 against a database consisting of 5527 non-redundant (NR) *Plasmodium falciparum* 3D7 proteins (PlasmoDB-42 release), 36661 NR human proteins (NCBI, 2018-03-30 release), 419 usual contaminants (human keratins, IgGs, and proteolytic enzymes). To estimate false discovery rates (FDRs), the amino acid sequence of each NR protein entry was randomized, which resulted in a total search space of 85246 NR sequences. All cysteines were considered as fully carboxamidomethylated (+57 Da statically added), while methionine oxidation was searched as a differential modification. DTASelect^73^ v.1.9 and swallow v.0.0.1, an in-house developed software (https://github.com/tzw-wen/kite)^74^, were used to filter ProLuCID search results at given FDRs at the spectrum, peptide, and protein levels. Here, all controlled FDRs were less than 1.2%. All datasets were contrasted against their merged data set, respectively, using Contrast v1.9 and in-house developed sandmartin v.0.0.1 (https://github.com/tzw-wen/kite/tree/master/kitelinux)^74^. Our in-house developed software, NSAF7 v.0.0.1 (https://github.com/tzw-wen/kite/tree/master/windowsapp/NSAF7x64)^74^, was used to generate spectral count-based label free quantitation results^75^. QSPEC^76^ was used to calculate log2 FC and *p* values to statistically compare *Pf*RAP01 and *Pf*RAP21 purifications to negative controls. Proteins with log2 FC ≥ 1 and *p* ≤ 0.05 were considered significantly enriched in the RAP purifications (Supplementary Data 5).

### eCLIP-seq

Late asexual parasites (7.5 × 10^9^ to 1.5 × 10^10^ cells) were extracted by saponin lysis and were crosslinked on ice by 254 nm UV light for a total of 1200 mJ/cm² with 2 min breaks using Spectrolinker^TM^ XL-1000. Ten μg of rabbit polyclonal HA tag antibody (abcam, ab9110) were coupled with Dynabeads^TM^ M-280 Sheep Anti-Rabbit IgG (Thermo Fisher, 11203D) for 1 h at room temperature. The following steps were processed using eCLIP Library Prep Kit (Eclipse BioInnovations, ECEK-0001) according to the manufacturer’s instructions. Briefly, cells were resuspended in 1 ml of lysis buffer (50 mM Tris-HCl pH 7.4, 100 mM NaCl, 1% NP-40, 0.1% SDS, 0.5% sodium deoxycholate, EDTA-free protease inhibitor cocktail (Roche, 11873580001) and 10 µL of Murine RNase inhibitor (NEB) in nuclease free water) and genomic DNA was sheared by sonication (Covaris ultrasonicator; 5 min, 5 % duty cycle, 140 intensity peak incident power, 200 cycles per burst). After RNA fragmentation with 100 units of RNase-I (Ambion, AM2294) for 5 min at 37 °C, lysates were incubated with antibody-coupled magnetic beads at 4 °C overnight. Two percent of each sample were saved prior to washes and correspond to negative control (Input). Immunoprecipitated (IP) samples were washed with high salt buffer (50 mM Tris-HCl pH 7.4, 1 M NaCl, 1 mM EDTA, 1% NP-40, 0.1% SDS, 0.5% sodium deoxycholate in nuclease free water) then with wash buffer (20 mM Tris-HCl pH 7.4, 10 mM MgCl2, 0.2% Tween 20, in nuclease free H2O). 5′ and 3′ RNA ends were repaired with PSP and PNK enzymes, followed by RNA adapter ligation. Protein-RNA complexes from IP and Input samples were eluted in loading buffer, separated by gel electrophoresis and transferred onto a nitrocellulose membrane at 4 °C overnight. The region comprising the protein band of interest up to an additional 75 kDa above (Parental samples: 40–150 kDa; *Pf*RAP21: 40–150 kDa; *Pf*RAP21: 60–175 kDa) were isolated and digested with proteinase K at 37 °C for 20 min then 50 °C for 20 min with interval mixing at 1200 rpm. RNA was cleaned and concentrated using Zymo RNA Clean & Concentrator kit (Zymo Research, R1015) and was reverse transcribed with AffinityScript enzyme (Agilent) at 54 °C for 20 min. After cDNA end repair, a 3’ ssDNA adapter was ligated, and qPCR was performed on Bio-Rad CFX Connect system. Libraries were amplified according to the Ct values obtained. PCR conditions consisted of 98 °C (30 s) followed by 6 cycles of (98 °C (15 s), 70 °C (30 s), 72 °C (40 s)), then (Ct-5) cycles of (98 °C (15 s), 72 °C (45 s)) and 72 °C (1 min). Libraries were loaded into a 3% agarose gel and regions between 175–350 bp were extracted and purified using MinElute Gel Extraction Kit (Qiagen, 28604) (Supplementary Fig. 7b). The quantity and quality of the final libraries were assessed using a Bioanalyzer (Agilent Technology Inc). All samples were multiplexed and sequenced by dual indexed run (PE100) on the Illumina NovaSeq 6000 sequencer at the UC San Diego IGM Genomics Center to produce 6 million reads per sample (Supplementary Data 9). Only forward reads were used for the computational analysis. FastQC^54^ was used to assess raw read quality. The 10-bp random-mer sequences at the beginning of the forward reads allowed the use of Clumpify (BBTools)^77^ to remove PCR duplicates. The random-mer sequences as well as adapter sequences were then removed using Trimmomatic^55^. Tails of reads were trimmed using Sickle^56^ with a Phred base quality threshold of 20, and reads shorter than 18 bp were removed. Remaining reads were aligned to the *P. falciparum* genome using Bowtie2 (version 2.3.4.1)^51^ with default parameters. Reads with a mapping quality score below 40 were removed using Samtools^52^ (Supplementary Data 9). The resulting BAM files were converted to BED by bedtools bamtobed, and genome-wide per-nucleotide read counts were obtained using bedtools genomecov with the -d parameter^58^. To normalize, all read counts were divided by the number of millions of mapped reads for each particular sample. At each nucleotide across the genome, the read counts for each IP sample were then subtracted by the read counts for the corresponding input sample, with negative values being converted to 0. For visualization in IGV^53^, these final normalized counts were converted to WIG files by a custom Python script, then to TDF files by igvtools totdf. To show strand differences, two BAM files were made from each BAM file after mapping quality filtering, one with only positive-strand reads, and one with only negative-strand reads. This was done using Samtools. Each BAM file was carried through the remaining steps, and the two resulting TDF files were combined into one IGV track. Peak calling was performed using MACS2 with the options --nomodel --extsize 150 --max-gap 1 -q 0.01. MACS2 was run without modeling due to the lack of a sufficient number of highly significant peaks for the model to be constructed. For each RAP protein, two IP samples were combined as the treatment reads, and two Input samples were combined as the control reads. Sequence reads have been deposited in the NCBI Sequence Read Archive with accession number PRJNA690830.

### Mitochondria enrichment

Isolation of mitochondria from *Pf*RAP01 and *Pf*RAP21 transgenic lines was performed as previously described with slight modifications^78^. Briefly, after 72 h of culture in presence of absence of aTc, ~10^10^ parasites were extracted by saponin treatment in 120 mM KCl, 20 mM NaCl, 20 mM Glucose, 6 mM HEPES, 6 mM MOPS, 1 mM MgCl_2_, 0.1 mM EGTA, and pH 7. After a final wash, cells were resuspended in 225 mM D-Mannitol (Sigma-Aldrich, M4125), 5 mM Succinic acid (Sigma-Aldrich, S3674), 5 mM L-(−)-Malic acid (Sigma-Aldrich, M1000), 75 mM Sucrose, 4.3 mM MgCl_2_, 10 mM Tris, 0.25 mM EGTA, 15 mM HEPES, and pH 7.6. The parasites were disrupted by N_2_ cavitation after pressurization at 1000 psi for 20 min at 4 °C using a Cell Disruption Vessel (Parr Instrument Company, 4635). Cell debris were removed by centrifugation at 900 × *g* for 6 min at 4 °C and the supernatants were passed through LS Columns (Miltenyi Biotec, 130-042-401). After centrifugation at 23,000 × *g* for 20 min at 4 °C, the pellets were resuspended in TRIzol® LS Reagent (Invitrogen, 10296028).

### Small RNA-sequencing

Mitochondrial and Total RNAs were purified as described above. RNA was first clean-up with Agencourt RNAclean XP beads (Beckman Coulter, A63987) and 1 µg of RNA was used for library preparation using the NEBNext Multiplex Small RNA Library Prep Set for Illumina (NEB E7300S/L). The following steps were processed according to the manufacturer’s instructions. After PCR amplification (94 °C 30 s, followed by 12 cycles of 94 °C 15 s, 62 °C 30 s, 70 °C 15 s, then 70 °C for 5 min), a size selection was performed on a 6% TBE PAGE gel. cDNA fragments between ~145–350 bp were isolated and eluted. After ethanol precipitation, the libraries were assessed on a Bioanalyzer (Agilent Technology Inc). All samples were multiplexed and sequenced on 100 nucleotides paired-end run on the Illumina NovaSeq 6000 sequencer at the UC San Diego IGM Genomics Center to produce at least 10 million of reads per sample (Supplemental Data 9).

FastQC^54^ was used to assess raw read quality. While sequencing was paired-end, only forward reads were used in this analysis. Adapter sequences were removed using Trimmomatic^55^. Tails of reads were trimmed using Sickle^56^ with a Phred base quality threshold of 20, and reads shorter than 15 bp were removed. These reads were then aligned to the *P. falciparum* genome (version 48, http://plasmodb.org) using Bowtie2^51^ with seed length 15 (-L 15). Only reads with a mapping quality score of 40 or higher (high-quality unique alignments) were retained, with filtering done using Samtools^52^. Raw read counts were determined for each gene in the *P. falciparum* genome using BedTools^58^ multicov to intersect the aligned reads with the genome annotation. Read counts were RPM-normalized by dividing by the number of millions of mapped reads for each library. R package pheatmap^60^ was used to generate heatmaps.

### Statistics

Two-tailed Mann–Whitney *U* test was performed on co-localization quantification. Parasitemia and proportion of asexual stages were analyzed using a two-way ANOVA with Tukey’s test for multiple comparisons. Replenishment of aTc was analyzed using a one-way ANOVA with Holm-Šídák correction. For DESeq2, the *p* values were obtained by two-tailed Wald test are corrected for multiple testing using the Benjamini–Hochberg correction. The GO terms enrichment analysis was determined using weight01 Fisher test. Two-tailed *t*-test was performed on metabolomics data. For the proteomics, the significance analysis was performed using QSPEC. MACS2 calculates a *p* value for each peak using Poisson distribution and *q* values are calculated using the Benjamini–Hochberg correction. Significant differences were indicated as following: * for *p* < 0.05; ** for *p* < 0.01, *** for *p* < 0.001 and **** for *p* < 0.0001. Statistical tests were performed with GraphPad Prism 6.

### Reporting summary

Further information on research design is available in the Nature Research Reporting Summary linked to this article.

## Supplementary information

Supplementary Information

Description of Additional Supplementary Files

Supplementary Data 1

Supplementary Data 2

Supplementary Data 3

Supplementary Data 4

Supplementary Data 5

Supplementary Data 6

Supplementary Data 7

Supplementary Data 8

Supplementary Data 9

Reporting Summary

## Data Availability

WGS, RNA-seq, eCLIP-seq, and small RNA-seq datasets generated in this study have been deposited in the NCBI Sequence Read Archive under accession number PRJNA690830. The MS datasets have been deposited in the ProteomeXChange (PXD023308) via the MassIVE repository (MSV000086636 with [10.25345/C5R795]), and may also be accessed from the Stowers Original Data Repository (http://www.stowers.org/research/publications/libpb-1571). The metabolomics data generated in this study have been deposited in the PanoramaWeb [https://panoramaweb.org/Plasmodium_RAPprotein.url]. Source data are provided with this paper.

## Source data

Source Data

## Code availability

The custom Python scripts used for eCLIP analysis have been previously published^79^. The entire in-house software suite (Kite) used for the MudPIT mass spectrometry analysis is available in Zenodo (10.5281/zenodo.5914885)^74^.
