# Supplementary material for: Functional genomics of RAP proteins and their role in mitoribosome regulation in Plasmodium falciparum
Source: Nat Commun. 2022 Mar 11;13:1275. doi: 10.1038/s41467-022-28981-7 (PMC8917122; doi:10.1038/s41467-022-28981-7)
Supplement: Supplementary file 1 — Supplementary Information [file 41467_2022_28981_MOESM1_ESM.pdf]

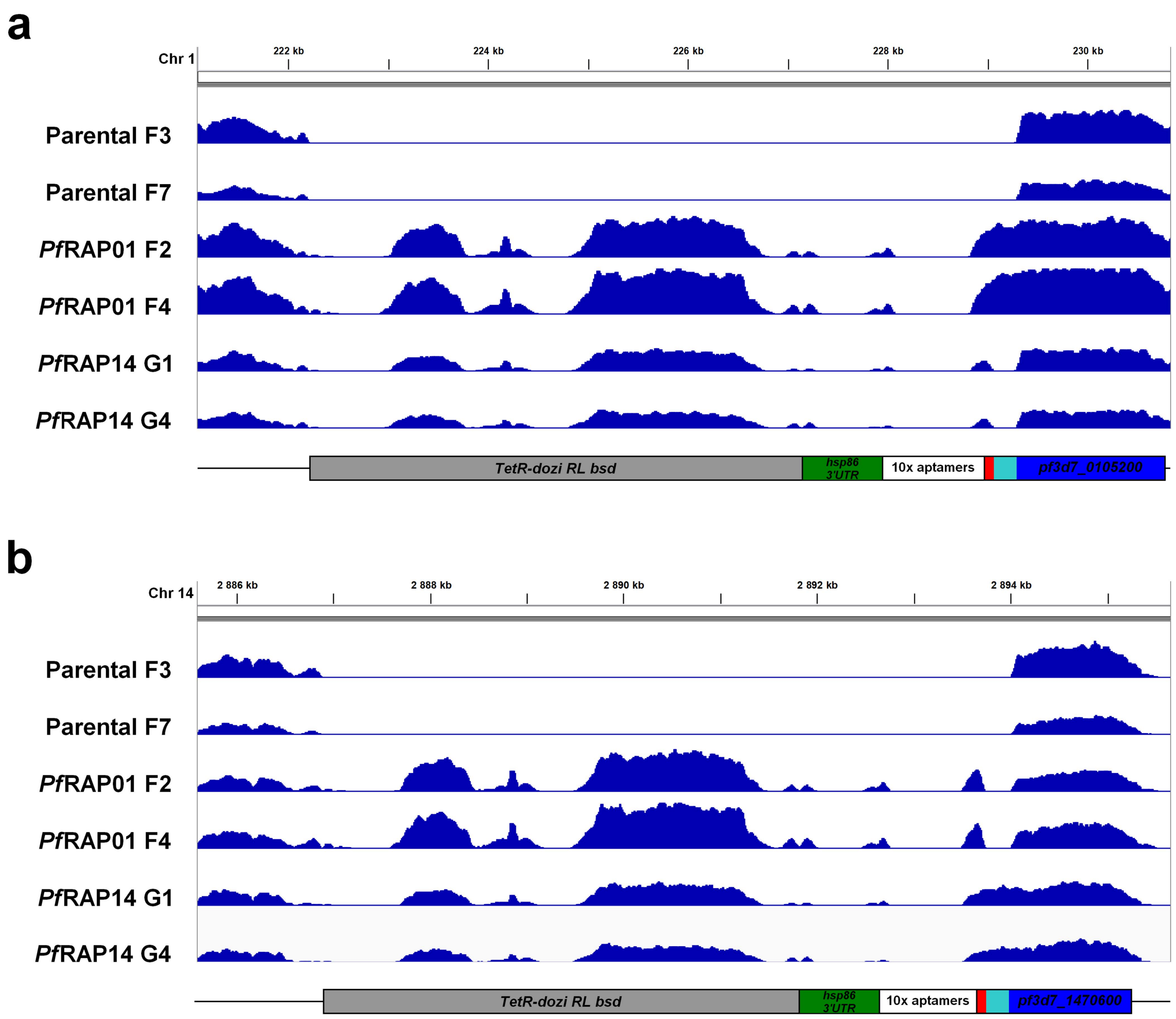

Supplementary Fig. 1: Integration of the inducible system in *Pf*RAP01 and *Pf*RAP21 locus. Read density tracks from the whole genome sequencing of parental, *Pf*RAP01, and *Pf*RAP21 clones. Reads are mapped on *pf3d7_0105200* (*Pf*RAP01) (**a**) and *pf3d7_1470600* (*Pf*RAP21) (**b**) loci. Schematic representation indicates the position of TetR-dozi-BSD cassette (grey), *hsp86* 3'UTR (green), 10x aptamers (white), HA-tag (red), recodonized sequences (light blue) and non-recodonized sequences (blue). TetR-dozi-BSD reads from the *Pf*RAP01 clones were also able to map on the modified chromosome 14 and vice versa with those from the *Pf*RAP21 clones. Reads from recodonized regions are detected only in their respective transgenic lines.

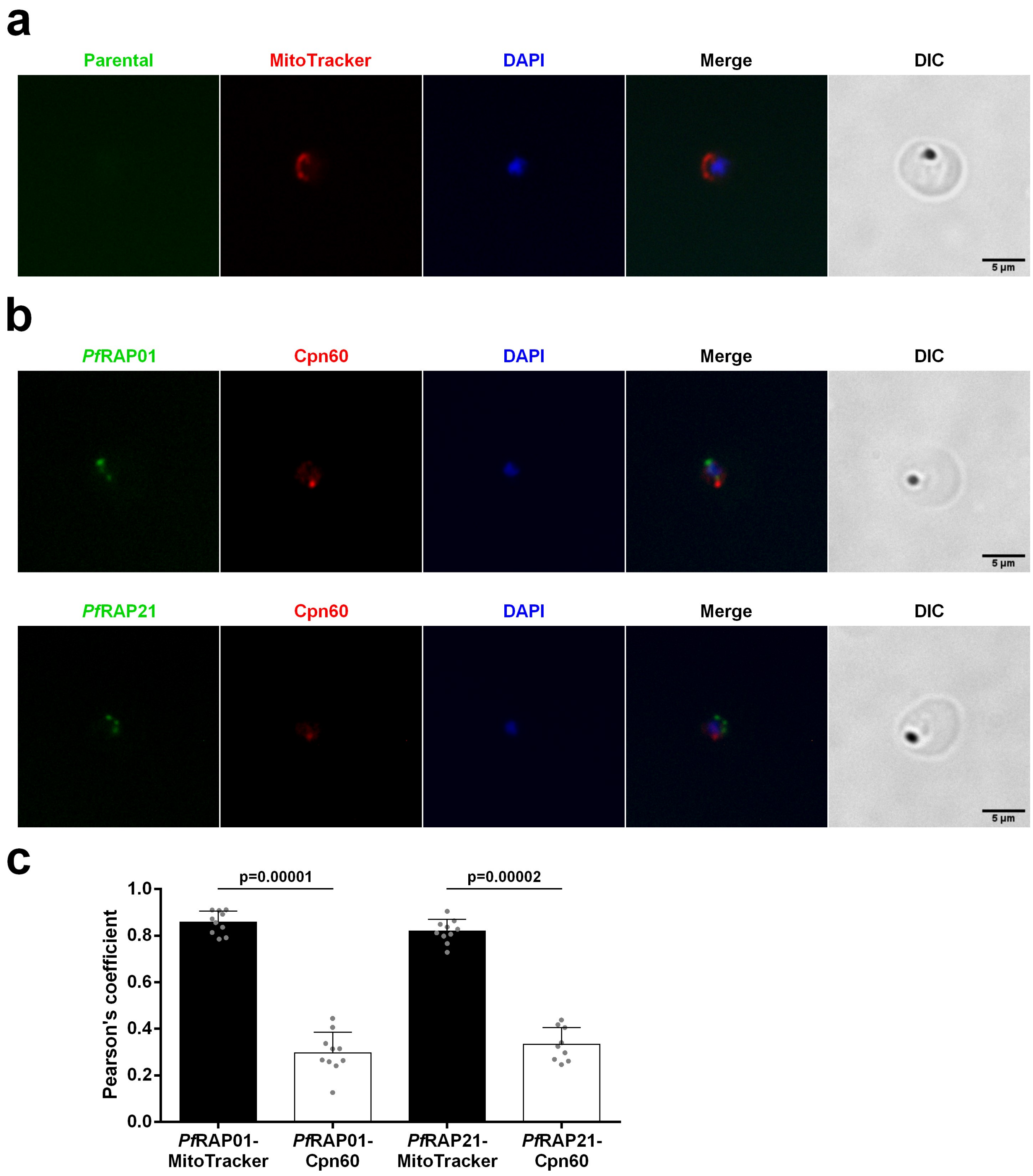

Supplementary Fig. 2: Immunofluorescence assays of parental, *Pf*RAP01, and *Pf*RAP21 lines. a. Immunofluorescence assay of parental parasites stained with anti-HA (green), MitoTracker (red), and DAPI (blue). Merge shows HA, MitoTracker, and DAPI signals. This IFA is representative of the localization observed on n>25 parasites from two independent experiments. **b.** Immunofluorescence assays of *Pf*RAP01 and *Pf*RAP21 parasites using anti-HA (green), anti-Cpn60 (red), and DAPI (blue). DIC: Differential Interference Contrast. **c.** Co-localizations of *Pf*RAP01 and *Pf*RAP21 HA-signals with MitoTracker or Cpn60 were quantified by measuring the Pearson correlation coefficient (mean ± SD) using JACoP. Each dot represents a Pearson correlation coefficient performed on a random parasite (n = 10 parasites) (Mann-Whitney U Test, two-tailed).

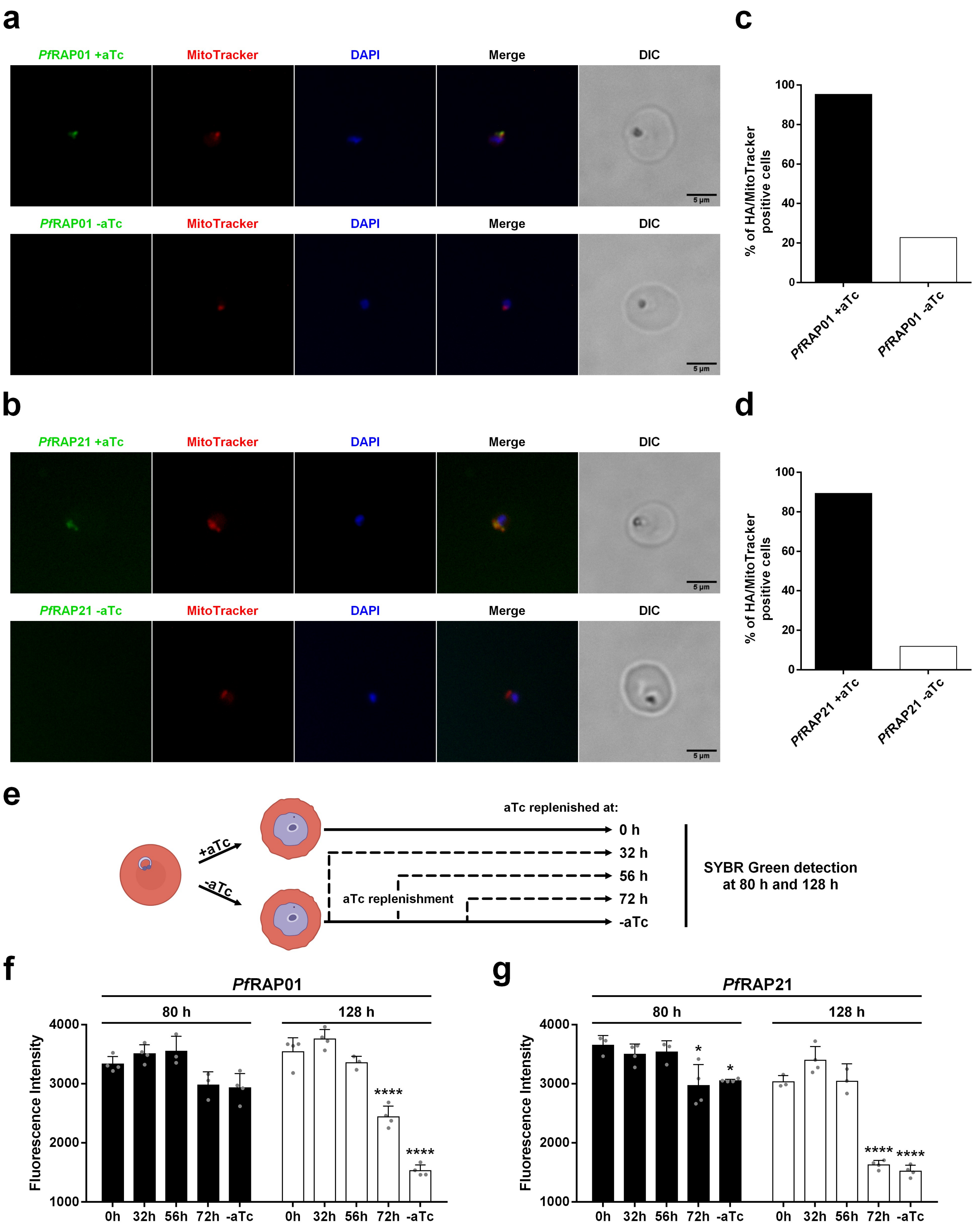

Supplementary Fig. 3: Effects of *Pf*RAP01 and *Pf*RAP21 knockdowns. Immunofluorescence assay of *Pf*RAP01 (**a**) and *Pf*RAP21 (**b**) ± aTc at 72 h. Double positive parasites for HA and MitoTracker signals were counted for *Pf*RAP01 (**c**) and *Pf*RAP21 (**d**) knockdowns and normalized by the total number of MitoTracker positive cells (n>50). DIC: Differential Interference Contrast. **e.** Schematic representation of aTc replenishment in *Pf*RAP01 and *Pf*RAP21 transgenic lines. Created with BioRender.com. DNA

quantification using SYBR Green of *Pf*RAP01 (**f**) and *Pf*RAP21 (**g**) cultures at 80 h and 128 h. The results represent the means ± SD of fluorescence intensities measured from two independent experiments in duplicate (n = 3-4). Each dot represents a fluorescence intensity. (One-way ANOVA with Holm-*Šídák* correction, * $p<0.05$ and **** $p<0.0001$ compared to condition 0 h).

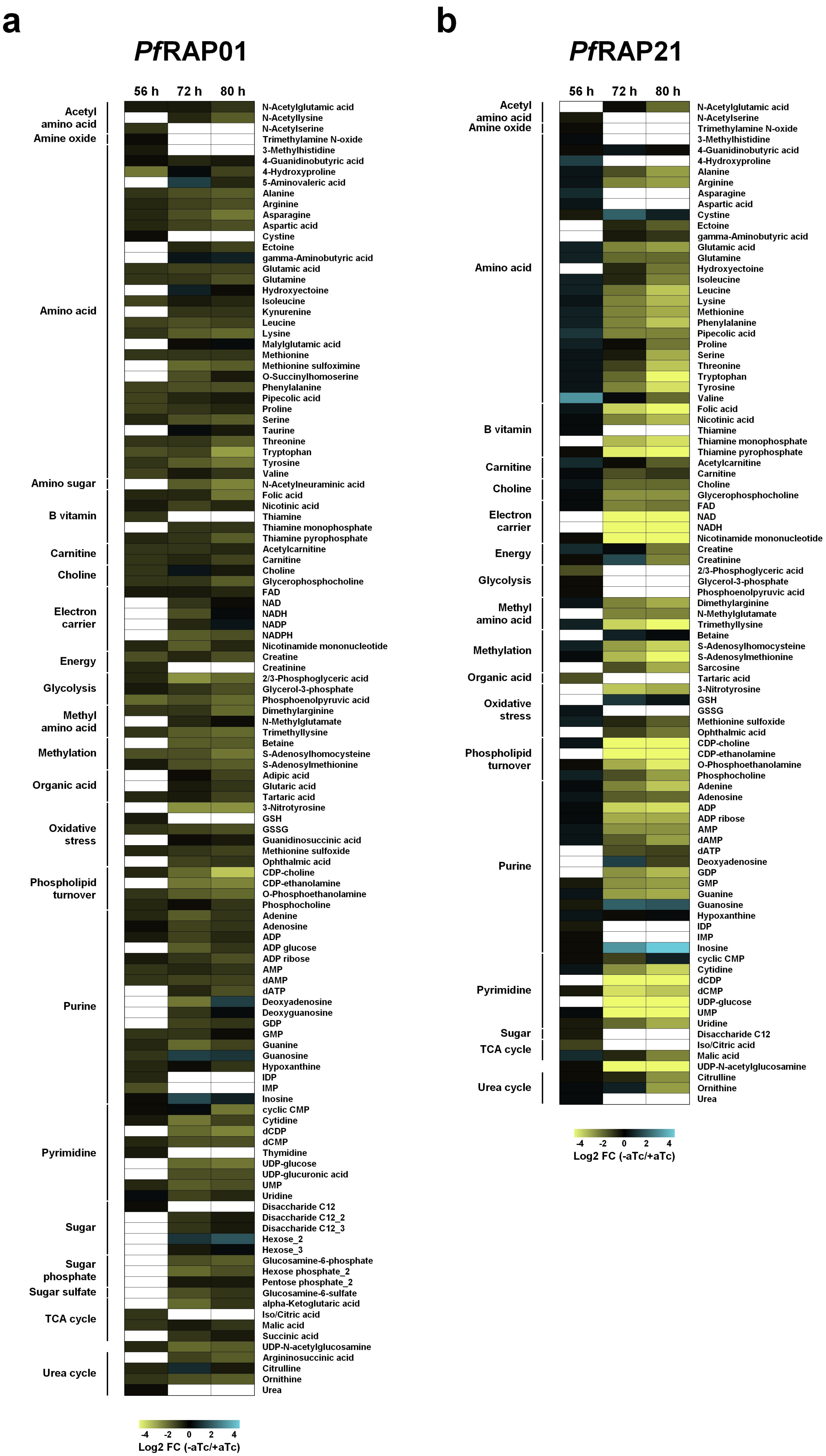
a
PfRAP01
56 h
72 h
80 h
Acetyl amino acid
Amine oxide
Amino acid
Amino sugar
B vitamin
Carnitine
Choline
Electron carrier
Energy
Glycolysis
Methyl amino acid
Methylation
Organic acid
Oxidative stress
Phospholipid turnover
Purine
Pyrimidine
Sugar
Sugar phosphate
Sugar sulfate
TCA cycle
Urea cycle
N-Acetylglutamic acid
N-Acetyllysine
N-Acetylserine
Trimethylamine N-oxide
3-Methylhistidine
4-Guanidinobutyric acid
4-Hydroxyproline
5-Aminovaleric acid
Alanine
Arginine
Asparagine
Aspartic acid
Cystine
Ectoine
gamma-Aminobutyric acid
Glutamic acid
Glutamine
Hydroxyectoine
Isoleucine
Kynurenine
Leucine
Lysine
Malylglutamic acid
Methionine
Methionine sulfoximine
O-Succinylhomoserine
Phenylalanine
Pipecolic acid
Proline
Serine
Taurine
Threonine
Tryptophan
Tyrosine
Valine
N-Acetylneuraminic acid
Folic acid
Nicotinic acid
Thiamine
Thiamine monophosphate
Thiamine pyrophosphate
Acetylcarnitine
Carnitine
Choline
Glycerophosphocholine
FAD
NAD
NADH
NADP
NADPH
Nicotinamide mononucleotide
Creatine
Creatinine
2/3-Phosphoglyceric acid
Glycerol-3-phosphate
Phosphoenolpyruvic acid
Dimethylarginine
N-Methylglutamate
Trimethyllysine
Betaine
S-Adenosylhomocysteine
S-Adenosylmethionine
Adipic acid
Glutaric acid
Tartaric acid
3-Nitrotyrosine
GSH
GSSG
Guanidinosuccinic acid
Methionine sulfoxide
Ophthalmic acid
CDP-choline
CDP-ethanolamine
O-Phosphoethanolamine
Phosphocholine
Adenine
Adenosine
ADP
ADP glucose
ADP ribose
AMP
dAMP
dATP
Deoxyadenosine
Deoxyguanosine
GDP
GMP
Guanine
Guanosine
Hypoxanthine
IDP
IMP
Inosine
cyclic CMP
Cytidine
dCDP
dCMP
Thymidine
UDP-glucose
UDP-glucuronic acid
UMP
Uridine
Disaccharide C12
Disaccharide C12_2
Disaccharide C12_3
Hexose_2
Hexose_3
Glucosamine-6-phosphate
Hexose phosphate_2
Pentose phosphate_2
Glucosamine-6-sulfate
alpha-Ketoglutaric acid
Iso/Citric acid
Malic acid
Succinic acid
UDP-N-acetylglucosamine
Argininosuccinic acid
Citrulline
Ornithine
Urea
-4 -2 0 2 4
Log2 FC (-aTc/+aTc)
b
PfRAP21
56 h
72 h
80 h
Acetyl amino acid
Amine oxide
Amino acid
B vitamin
Carnitine
Choline
Electron carrier
Energy
Glycolysis
Methyl amino acid
Methylation
Organic acid
Oxidative stress
Phospholipid turnover
Purine
Pyrimidine
Sugar
TCA cycle
Urea cycle
N-Acetylglutamic acid
N-Acetylserine
Trimethylamine N-oxide
3-Methylhistidine
4-Guanidinobutyric acid
4-Hydroxyproline
Alanine
Arginine
Asparagine
Aspartic acid
Cystine
Ectoine
gamma-Aminobutyric acid
Glutamic acid
Glutamine
Hydroxyectoine
Isoleucine
Leucine
Lysine
Methionine
Phenylalanine
Pipecolic acid
Proline
Serine
Threonine
Tryptophan
Tyrosine
Valine
Folic acid
Nicotinic acid
Thiamine
Thiamine monophosphate
Thiamine pyrophosphate
Acetylcarnitine
Carnitine
Choline
Glycerophosphocholine
FAD
NAD
NADH
Nicotinamide mononucleotide
Creatine
Creatinine
2/3-Phosphoglyceric acid
Glycerol-3-phosphate
Phosphoenolpyruvic acid
Dimethylarginine
N-Methylglutamate
Trimethyllysine
Betaine
S-Adenosylhomocysteine
S-Adenosylmethionine
Sarcosine
Tartaric acid
3-Nitrotyrosine
GSH
GSSG
Methionine sulfoxide
Ophthalmic acid
CDP-choline
CDP-ethanolamine
O-Phosphoethanolamine
Phosphocholine
Adenine
Adenosine
ADP
ADP ribose
AMP
dAMP
dATP
Deoxyadenosine
GDP
GMP
Guanine
Guanosine
Hypoxanthine
IDP
IMP
Inosine
cyclic CMP
Cytidine
dCDP
dCMP
UDP-glucose
UMP
Uridine
Disaccharide C12
Iso/Citric acid
Malic acid
UDP-N-acetylglucosamine
Citrulline
Ornithine
Urea
-4 -2 0 2 4
Log2 FC (-aTc/+aTc)

Supplementary Fig. 4: Metabolic analysis of *Pf*RAP01 and *Pf*RAP21 knockdown lines. Heatmap of polar metabolites in *Pf*RAP01 (**a**) and *Pf*RAP21 (**b**) parasites at 56 h, 72 h, and 80 h. The rows show the respective polar metabolites which are grouped according to their class. Colors represent log2 FC values for relative abundances between -aTc and +aTc samples. Blue and yellow indicate up- and down-regulated metabolites in RAP-deficient parasites, respectively. Missing values are shown in white.

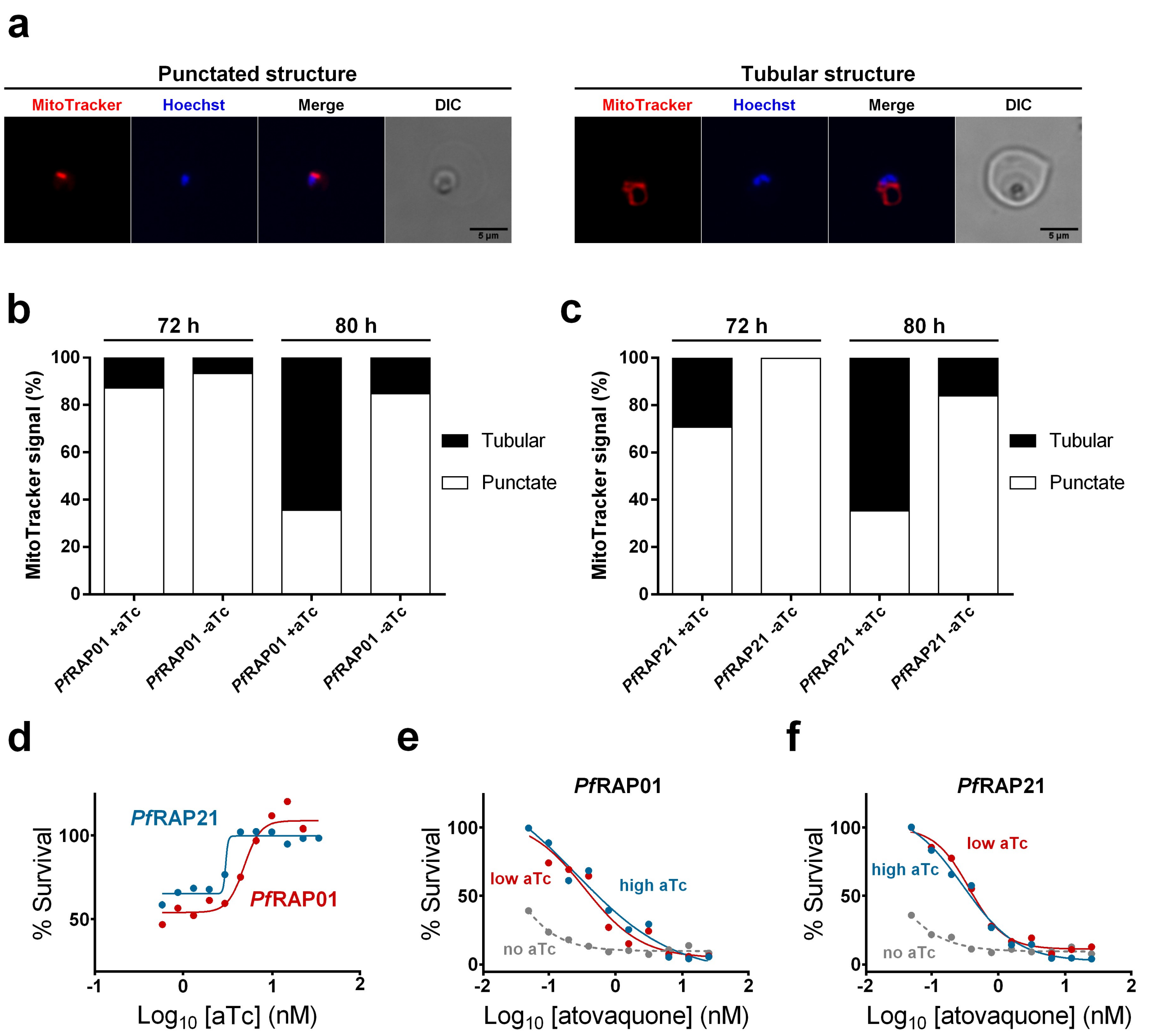

Supplementary Fig. 5: Impact of *Pf*RAP01 and *Pf*RAP21 knockdowns on parasite mitochondrion. a. Live-cell imaging assays of RAP parasites with MitoTracker and Hoechst 33342. DIC: Differential Interference Contrast. (**b-c**). Quantification of mitochondria showing punctuated or tubular structures using Mitotracker staining in *Pf*RAP01 (**b**) and *Pf*RAP21 (**c**) lines at 72 h and 80 h (n>30). (**d-f**). Comparison of dose-response curves of aTc (**d**) and atovaquone in *Pf*RAP01 (**e**) and *Pf*RAP21 (**f**) lines with and without aTc for 72 h. Data shown are the mean of duplicates and are representative of n = 2 and 3 independent experiments for **d-e** and **f**, respectively.

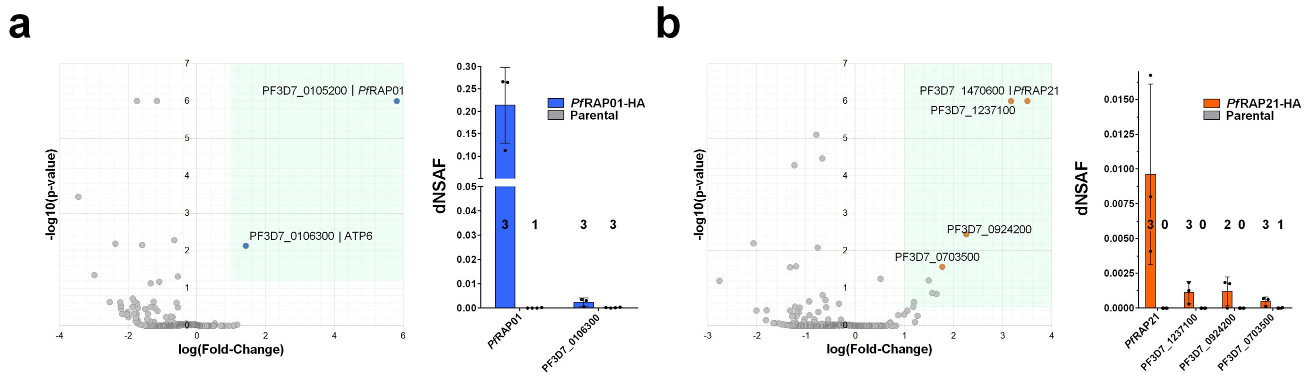

Supplementary Fig. 6: Immunoprecipitation of *Pf*RAP01 and *Pf*RAP21 proteins. Identified proteins after *Pf*RAP01-HA (**a**) and *Pf*RAP21-HA (**b**) immunoprecipitations. Volcano significance plots highlight proteins significantly enriched in the RAP affinity purifications compared to control purifications from the parental strain (QSPEC-calculated Log FC ≥1 and p-value ≤0.05, green area). Three immunoprecipitations were performed each for *Pf*RAP01-HA and *Pf*RAP21-HA along with 4 negative controls experiments (Supplementary Data 5). The relative abundance of bait proteins (*Pf*RAP01 and *Pf*RAP21) and their putative interaction partners is plotted as averaged dNSAF ± SD, with the number of times each protein was detected by experiment type reported as numbers above (or within) the bars. Each dot represents the dNSAF value for each replicate.

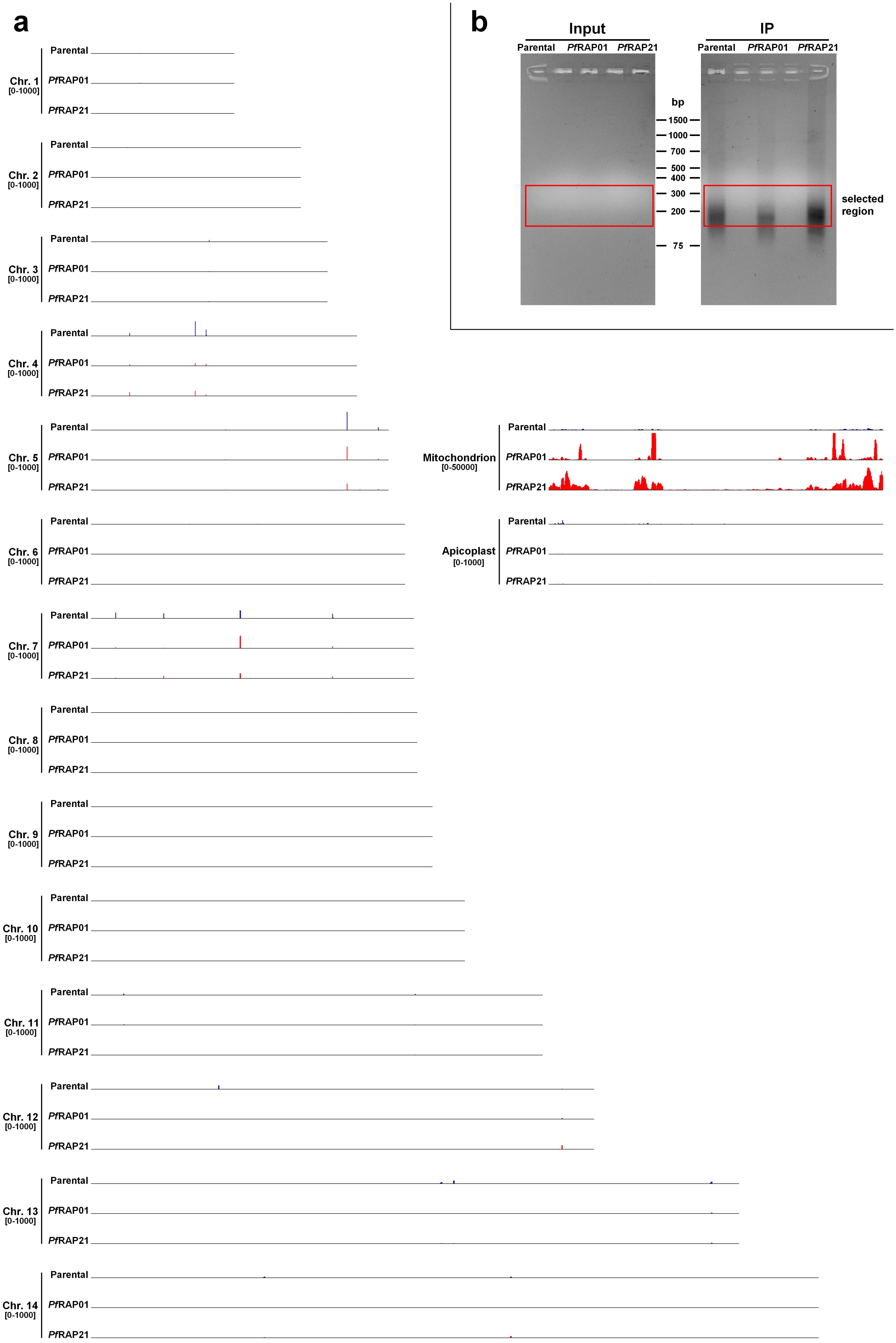
a
Chr. 1
[0-1000]
Chr. 2
[0-1000]
Chr. 3
[0-1000]
Chr. 4
[0-1000]
Chr. 5
[0-1000]
Chr. 6
[0-1000]
Chr. 7
[0-1000]
Chr. 8
[0-1000]
Chr. 9
[0-1000]
Chr. 10
[0-1000]
Chr. 11
[0-1000]
Chr. 12
[0-1000]
Chr. 13
[0-1000]
Chr. 14
[0-1000]
Parental
PfRAP01
PfRAP21
Mitochondrion
[0-50000]
Apicoplast
[0-1000]
b
Input
IP
Parental
PfRAP01
PfRAP21
bp
1500
1000
700
500
400
300
200
75
selected region

Supplementary Fig. 7: eCLIP-seq analysis of *Pf*RAP01 and *Pf*RAP21 proteins. a. Read density tracks along the 14 chromosomes, apicoplast, and mitochondrial genome for parental, *Pf*RAP01, and *Pf*RAP21 samples. Values represented are an average of the two replicates. The scales were adjusted to provide the best possible visualization (0 to 1000 for nuclear and apicoplast genomes and 0 to 50000 for mitochondrial genome). The size of apicoplast and mitochondrial genomes is not to scale. **b**. Quality control check and size selection of eCLIP-seq libraries using 3% agarose gel. The gel shown is representative of two independent experiments performed.

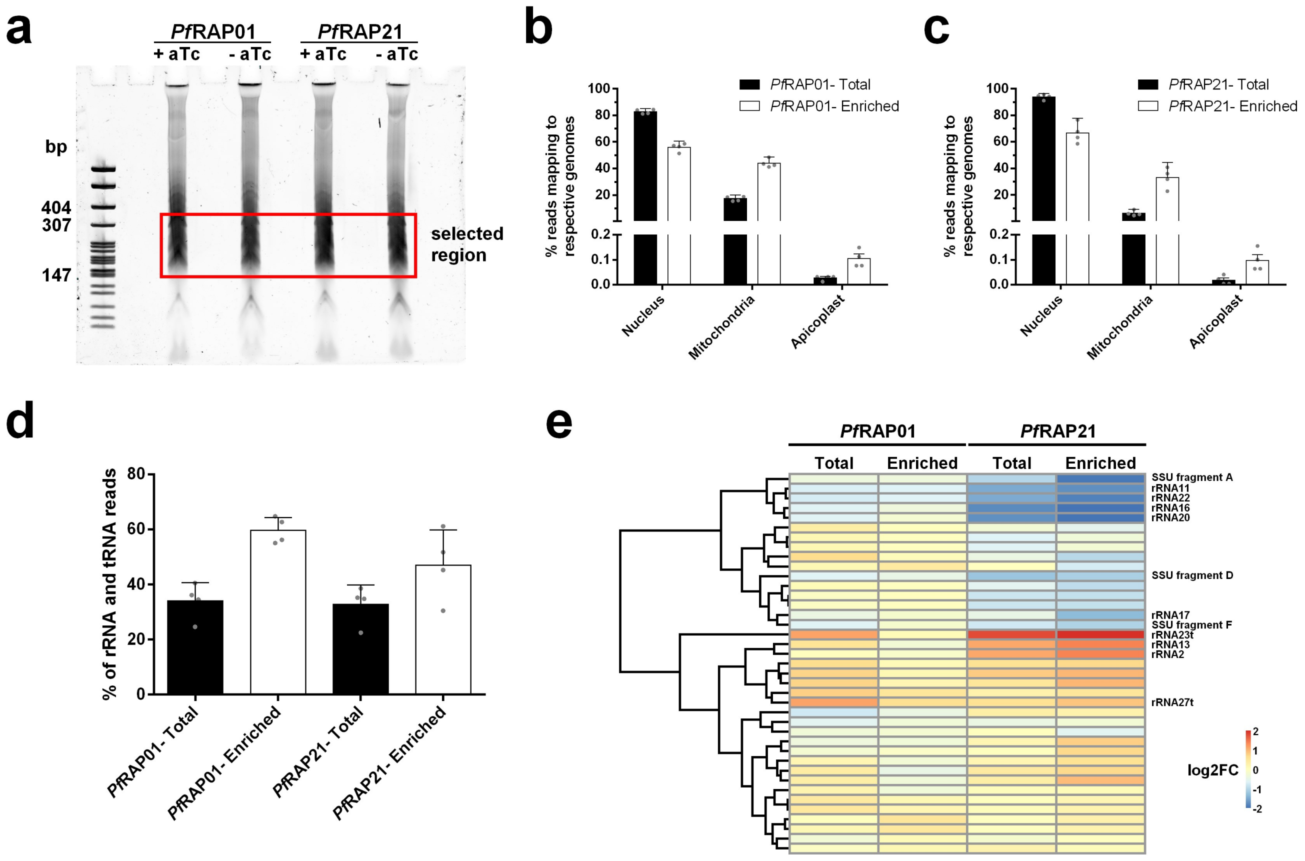

Supplementary Fig. 8: Small RNA-seq analysis of *Pf*RAP01 and *Pf*RAP21 knockdowns. a. Quality control check and size selection of small RNA-seq libraries using 6% polyacrylamide gel. The gel shown is representative of the experiments performed. (**b-c**). Distribution of *Pf*RAP01 (**b**) and *Pf*RAP21 (**c**) reads to the nuclear, apicoplast, and mitochondrial genome. Bars represented average percentages of each replicate ± SD from total and enriched RNA samples. Two independent experiments were performed with and without aTc for each RNA isolation method. Each dot represents the percentage for each replicate. **d**. Average percentage ± SD of rRNA and tRNA reads in total and enriched RNA samples of *Pf*RAP01 and *Pf*RAP21. Two independent experiments were performed with and without aTc for each RNA isolation method. Each dot represents the percentage for each replicate. **e**. Heatmaps of the log2 FC values of mitochondrial rRNA from total and enriched RNA samples of *Pf*RAP01 and *Pf*RAP21. Gene identifiers are indicated for the rRNAs with log2 FC >1 or <1 in at least one condition.
