# Supplementary material for: Functional genomics of RAP proteins and their role in mitoribosome regulation in Plasmodium falciparum
Source: Nat Commun. 2022 Mar 11;13:1275. doi: 10.1038/s41467-022-28981-7 (PMC8917122; doi:10.1038/s41467-022-28981-7)
Supplement: Supplementary file 2 — Description of Additional Supplementary Files [file 41467_2022_28981_MOESM2_ESM.pdf]

Description of Additional Supplementary Files

File Name: Supplementary Data 1
Description: **List of predicted RAP proteins in P. falciparum.** The table regroups the RAP proteins identified in P. falciparum 3D7 and indicates the PlasmoDB identifier, gene annotation, protein length and the predicted position of the RAP domains for each protein. Recent large-scale genetic screenings were performed to study their essentiality and the results are reported here. The homologs of P. falciparum RAP proteins in P. berghei and P. vivax were provided by PlasmoDB.

File Name: Supplementary Data 2
Description: **Transcriptomics data obtained by RNA-seq from PfRAP01 and PfRAP21 knockdown parasites.** For each RAP protein, the list of genes used on the heatmap, DEseq2 results at the different time points, and raw datasets are presented in different sheets. Comparison results between the RAP +aTc lines and Parental line are also included along with Spearman correlation coefficients. The p-values were obtained by two-tailed Wald test and corrected for multiple testing using the Benjamini-Hochberg correction.

File Name: Supplementary Data 3
Description: **Metabolomics data obtained from PfRAP01 and PfRAP21 knockdown parasites**. Polar metabolites and lipidomics (compiled and raw data) are presented in different sheets for each RAP protein. Samples at 56 h were processed separately from the 72 h and 80 h samples. Pvalues from two-tailed t-test and log2 FC (- aTc/+aTc) are indicated for each time point. Metabolites are indicated as Up or Down if pvalues<-1 (blue) or log2 FC>1 (red) at 56 h or 72 h and 80 h. m/z= mass-to-charge ratio, RT= retention time, #N/A= metabolite not detected under both conditions for a given time point.

File Name: Supplementary Data 4
Description: **Compound susceptibility assays in PfRAP01 and PfRAP21 knockdowns.** Growth inhibition was analyzed after 72 h and 120 h using the Renilla-Glo(R) Luciferase Assay System. IC50 values were obtained from corrected doseresponse curves. ND=Not Determined.

File Name: Supplementary Data 5
Description: **Proteins identified by MudPIT analyses of the proteins co- purified with PfRAP01-HA, PfRAP21-HA, and negative controls.** The significance analysis was performed using QSPEC.

File Name: Supplementary Data 6
Description: **Peak calling of eCLIP-seq samples using MACS2.** For each RAP protein, two IP samples were combined as the treatment reads, and two Input samples were combined as the control reads. P-values were calculated for each peak using Poisson distribution and q-values using the BenjaminiHochberg correction.

File Name: Supplementary Data 7
Description: **Transcriptomics data obtained by small RNA-seq from PfRAP01 and PfRAP21 knockdown parasites.** Mitochondrial rRNAs only, all rRNAs and tRNAs, and raw dataset were presented in different sheets. Read counts were RPM-normalized by dividing by the number of millions of mapped reads for each library.

File Name: Supplementary Data 8
Description: **List of oligonucleotides**. The oligonucleotides were synthetized by IDT at 25 nmole scale.

File Name: Supplementary Data 9
Description: **Summary of sequencing data analysis.**
