# Supplementary material for: Functional genomics of RAP proteins and their role in mitoribosome regulation in Plasmodium falciparum
Source: Nat Commun. 2022 Mar 11;13:1275. doi: 10.1038/s41467-022-28981-7 (PMC8917122; doi:10.1038/s41467-022-28981-7)
Supplement: Supplementary file 12 — Reporting Summary [file 41467_2022_28981_MOESM12_ESM.pdf]

nature portfolio

Corresponding author(s): Karine Le Roch

Last updated by author(s): Feb 3, 2022

Reporting Summary

Nature Portfolio wishes to improve the reproducibility of the work that we publish. This form provides structure for consistency and transparency in reporting. For further information on Nature Portfolio policies, see our Editorial Policies and the Editorial Policy Checklist.

Statistics

For all statistical analyses, confirm that the following items are present in the figure legend, table legend, main text, or Methods section.

n/a	Confirmed	
☐	☒	The exact sample size (*n*) for each experimental group/condition, given as a discrete number and unit of measurement
☐	☒	A statement on whether measurements were taken from distinct samples or whether the same sample was measured repeatedly
☐	☒	The statistical test(s) used AND whether they are one- or two-sided<br>*Only common tests should be described solely by name; describe more complex techniques in the Methods section.*
☒	☐	A description of all covariates tested
☐	☒	A description of any assumptions or corrections, such as tests of normality and adjustment for multiple comparisons
☐	☒	A full description of the statistical parameters including central tendency (e.g. means) or other basic estimates (e.g. regression coefficient) AND variation (e.g. standard deviation) or associated estimates of uncertainty (e.g. confidence intervals)
☐	☒	For null hypothesis testing, the test statistic (e.g. *F*, *t*, *r*) with confidence intervals, effect sizes, degrees of freedom and *P* value noted<br>*Give P values as exact values whenever suitable.*
☒	☐	For Bayesian analysis, information on the choice of priors and Markov chain Monte Carlo settings
☒	☐	For hierarchical and complex designs, identification of the appropriate level for tests and full reporting of outcomes
☐	☒	Estimates of effect sizes (e.g. Cohen's *d*, Pearson's *r*), indicating how they were calculated

Our web collection on statistics for biologists contains articles on many of the points above.

Software and code

Policy information about availability of computer code

Data collection	Image Lab software (version 5)<br>Zeiss (version 2.3)<br><br>Metabolomics:<br>Progenesis Qi (version 2.4)<br>Skyline (version 20.1)
Data analysis	GraphPad Prism (version 6 and 8)<br>ImageJ (version 1.53i)<br><br>High-throughput sequencing:<br>Bowtie2 (version 2.3.4.1)<br>HISAT (version 2-2.1.0)<br>FastQC (version 0.11.8)<br>Trimmomatic (version 0.36)<br>Sickle (version 1.33)<br>BBTools (includes Clumpify) (version 38.87)<br>Bedtools (version 2.27.1)<br>Samtools (version 1.11)<br>DESeq2 (version 1.28.1)<br>IGV (version 2.7.2)<br>MACS2 (version 2.2.7.1)

R package pheatmap (version 1.0.12)

Metabolomics:
RAMClust (version 1.1.0)

Proteomics:
ProLuCID (version 1.3.3)
DTASelect (version 1.9)
Swallow (version 0.0.1) https://github.com/tzw-wen/kite
Contrast (version 1.9)
Sandmartin (version 0.0.1) https://github.com/tzw-wen/kite/tree/master/kitelinux
NSAF7 (version 0.0.1) https://github.com/tzw-wen/kite/tree/master/windowsapp/NSAF7x64

For manuscripts utilizing custom algorithms or software that are central to the research but not yet described in published literature, software must be made available to editors and reviewers. We strongly encourage code deposition in a community repository (e.g. GitHub). See the Nature Portfolio guidelines for submitting code & software for further information.

Data

Policy information about availability of data

All manuscripts must include a data availability statement. This statement should provide the following information, where applicable:

- Accession codes, unique identifiers, or web links for publicly available datasets
- A description of any restrictions on data availability
- For clinical datasets or third party data, please ensure that the statement adheres to our policy

WGS, RNA-seq, eCLIP-seq, and small RNA-seq datasets generated in this study have been deposited in the NCBI Sequence Read Archive under accession number PRJNA690830 [https://www.ncbi.nlm.nih.gov/bioproject/PRJNA690830]. The MS datasets have been deposited in the ProteomeXChange (PXD023308 [http://proteomecentral.proteomexchange.org/cgi/GetDataset?ID=PXD023308]) via the MassIVE repository (MSV000086636 [https://massive.ucsd.edu/ProteoSAFe/dataset.jsp?accession=MSV000086636] with [doi:10.25345/C5R795]), and may also be accessed from the Stowers Original Data Repository (http://www.stowers.org/research/publications/libpb-1571). Source data are provided as a Source Data file. The metabolomics data generated in this study have been deposited in the PanoramaWeb [https://panoramaweb.org/Plasmodium_RAPprotein.url].

Field-specific reporting

Please select the one below that is the best fit for your research. If you are not sure, read the appropriate sections before making your selection.

☒ Life sciences ☐ Behavioural & social sciences ☐ Ecological, evolutionary & environmental sciences

For a reference copy of the document with all sections, see nature.com/documents/nr-reporting-summary-flat.pdf

Life sciences study design

All studies must disclose on these points even when the disclosure is negative.

Sample size	Sample sizes were chosen according to standards in the field and based on our previous experience with similar experiments. For parasitemia, at least 10 fields were counted (300-500 red blood cells) and the phenotypic analyzes were assessed with at least 51 parasites (51 to 106 parasites). At least 25 parasites were observed for IFAs. Sample size and statistical analysis are indicated in the Methods section and Source Data.
Data exclusions	No data were excluded.
Replication	All attempts at replication were successful. Spearman correlation coefficient was performed to validate the reproducibility of RNA-seq and eCLIP-seq performed in duplicate. The metabolomics experiments were performed in triplicate and validated using PCA analysis for each replicate and each condition. IP-MS was performed in triplicate. Small RNA-seq experiments were performed in duplicate. Results generated with two different RNA isolation methods validated the robustness of this experiment. Parasitemia, phenotypic analysis, immunoblots, aTc replenishment and IFAs were done in replicate in at least two independent experiments.
Randomization	Assignment of parasites to treatment and control groups were randomized in the study.
Blinding	The parasitaemia and phenotypic analyzes were blind validated by a third person. Proteomics and metabolomics samples were blinded for our collaborators.

Reporting for specific materials, systems and methods

We require information from authors about some types of materials, experimental systems and methods used in many studies. Here, indicate whether each material, system or method listed is relevant to your study. If you are not sure if a list item applies to your research, read the appropriate section before selecting a response.

Materials & experimental systems

n/a	Involved in the study
☐	☒ Antibodies
☐	☒ Eukaryotic cell lines
☒	☐ Palaeontology and archaeology
☒	☐ Animals and other organisms
☒	☐ Human research participants
☒	☐ Clinical data
☒	☐ Dual use research of concern

Methods

n/a	Involved in the study
☒	☐ ChIP-seq
☒	☐ Flow cytometry
☒	☐ MRI-based neuroimaging

Antibodies

Antibodies used	Primary antibodies for IFA: Anti-HA mAb (Abcam, ab24779, [16B12], lot GR153254-8) at 1:500 and rabbit anti-Cpn60 (kindly provided by Dr. Boris Striepen) at 1:1000.<br>Secondary antibodies for IFA: Goat anti-Mouse Alexa Fluor 488 (Invitrogen, A11001, lot 2051236) at 1:2000 and Donkey anti-Rabbit Alexa Fluor 568 (Invitrogen, A10042, lot 685232) at 1:2000.<br>Primary antibodies for immunoblot: Rabbit anti-HA antibody (Abcam, ab9110, lot GR3320789-1) at 1:2500 and Anti-Plasmodium aldolase antibody (Abcam, ab207494) at 1:10000.<br>Secondary antbody for immunoblots: HRP-labeled Goat anti-Rabbit IgG (H+L) (NovexTM, A16104, lot 42-28-042114) at 1:10000.<br>Immunoprecipitation: Rabbit anti-HA antibody (Abcam, ab9110, lot GR3320789-1) at 1:100.
Validation	Anti-HA mAb (ab27779) and rabbit anti-HA (ab9110) were validated by Abcam for IFA, immunoblot and immunoprecipitation.<br>anti-Cpn60 was validated by IFA in Plasmodium falciparum in a previous publication: https://doi.org/10.1073/pnas.1919501117<br>Anti-Plasmodium aldolase antibody (ab207494) was validated by Abcam for immunoblot.

Eukaryotic cell lines

Policy information about cell lines

Cell line source(s)	Plasmodium falciparum NF54 strain was provided by the Malaria Research and Reference Reagent Resource Center (MR4)
Authentication	Lines were analyzed and validated by whole-genome sequencing
Mycoplasma contamination	Not tested
Commonly misidentified lines (See ICLAC register)	No commonly misidentified lines were used in this study
